# Association Between Self-Reported Snoring and Metabolic Syndrome: A Systematic Review and Meta-Analysis

**DOI:** 10.3389/fneur.2020.517120

**Published:** 2020-10-02

**Authors:** Jinsha Ma, Huifang Zhang, Hui Wang, Qian Gao, Heli Sun, Simin He, Lingxian Meng, Tong Wang

**Affiliations:** Department of Health Statistics, School of Public Health, Shanxi Medical University, Taiyuan, China

**Keywords:** snoring, metabolic syndrome, meta-analysis, dose-response, systematic review

## Abstract

**Background:** Snoring is a common condition. Previous studies have reported the relationships between snoring and metabolic syndrome (MetS) and/or its five components: hypertension, hyperglycemia, low-high density lipoprotein (low-HDL), high-triglyceride level, and abdominal obesity. However, conclusions have been inconsistent, and there has been no comprehensive summary on this. Therefore, we performed a systematic review on the relationships between snoring and MetS, including each of MetS' components.

**Methods:** A systematic review and a meta-analysis were conducted following the Meta-analysis of Observational Studies in Epidemiology group and Preferred Reporting Items for Systematic Reviews and Meta-analysis guidelines. Electronic databases including PubMed, Embase, and the Cochrane Library were searched for publications from inception to 15 July 2020. The inverse-variance weighted method was used in the meta-analysis to calculate the pooled odds ratios (ORs) and their 95% confidence intervals (CIs) to determine the association between snoring and MetS (and its components) through a fixed or random effect model. A restricted cubic spline regression model and the linear regression model were used in a two-stage dose–response meta-analysis to evaluate the non-linear and the linear trends between snoring frequency and MetS and its components.

**Results:** A total of 40 studies with 966,652 participants were included in this study. The pooled ORs between snoring and MetS and its components, hypertension, hyperglycemia, low-HDL, high-triglyceride level, and abdominal obesity, were 1.61 (95% CI, 1.43–1.78), 1.23 (95% CI, 1.15–1.31), 1.05 (95% CI, 1.04–1.07), 1.09 (95% CI, 1.00–1.18), 1.08 (95% CI, 1.00–1.17), and 1.75 (95% CI, 1.46–2.05), respectively. Non-linear trends were detected in the five associations except for low-HDL. A linear trend was detected in the association of snoring with hypertension, hyperglycemia, low-HDL, or abdominal obesity, with ORs of 1.07 (95% CI, 1.01–1.13), 1.05 (95% CI, 1.02–1.08), 1.03 (95% CI, 1.02–1.04), and 1.17 (95% CI, 1.16–2.89), respectively.

**Conclusion:** Snoring was a risk factor of MetS, and a dose–response relationship existed between the two. Timely intervention in identifying snorers can minimize as much as possible the risk of metabolic syndrome in those who snore.

## Introduction

Metabolic syndrome (MetS), a combination of abdominal obesity and abnormal blood pressure, lipid, and/or glycometabolism (1), is an established risk factor for many chronic diseases, such as prostatic hyperplasia (2), cardiovascular-related disease (3), and subsequent mortality (4). Given the prevalence of MetS in 20–25% of adults worldwide (5), identifying modifiable risk factors for the syndrome is of critical public health importance. Snoring, a physical phenomenon that occurs due to the high-frequency vibrations of the respiratory structures, was reported in some studies to be a pathophysiologic entity that increases the risk for MetS and/or its components (6–9) but was not reported as such in others (10, 11). Furthermore, even among studies reporting significant associations, the odds ratio (OR) magnitude varied substantially, even in studies that gave consistently significant conclusions, such as 1.38 [95% confidence interval (CI), 1.17–1.62] by Li et al. (8) and 2.252 (95% CI, 1.298–3.906) by Roopa et al. (9). Snoring is an easily measured and controllable factor, and if it is related to MetS and/or its components, the size of the correlation may provide insights into MetS prevention and management.

In view of such a situation, Xiong et al. (12) retrieved studies on a relationship between snoring and diabetes to perform a meta-analysis. However, their study identified the relationship between snoring frequency and risk of diabetes in the highest vs. the lowest categories of snoring frequency. Such a practice cannot make full use of the information of the included studies and may have generally exaggerated the associations between snoring and diabetes. Furthermore, Xiong et al. (12) summarized only one of MetS' components; thus, a comprehensive review of the relationship, especially one showing a dose–response trend, between snoring and MetS and each of its components is still necessary.

In this study, we performed a systematic review on the relationships between snoring and MetS and each of its five components. For studies reporting snoring as a dichotomous variable, we performed a typical meta-analysis, while a dose–response meta-analysis was performed for studies reporting snoring as a ranked variable in order to give more convincing conclusions.

## Materials and Methods

The present study was conducted in accordance with the guidelines of the Meta-analysis of Observational Studies in Epidemiology group (13) and Preferred Reporting Items for Systematic Reviews and Meta-analysis (14). This meta-analysis was registered in the PROSPERO platform successfully (CRD42020150070).

### Search Strategy

In this study, electronic databases (PubMed, Embase, and the Cochrane Library) were searched for publications prior to 15 July 2020. The following combinations of medical subject headings and text words were used to retrieve eligible studies: ((((((((((((((MS) or Mets) or X syndrome) or syndrome X) or metabolic syndrome) or metabolic syndrome X) or insulin resistance syndrome))) OR obesity) OR (((hypertension) or high blood pressure))) OR ((((((diabetes) or diabetic) or hyperglycemia) or diabetes mellitus) or high blood sugar))) OR (((((((((triglyceride) or hypertriglyceridemia) or HDL) or cholesterol) or hypercholesterolemia) or hyperlipidemia) or dyslipidemia))) OR (((cardiovascular risk) or cardiometabolic risk))) AND (snoring or snorer). We carefully read degree theses and conference papers before excluding them from this study and contacted the corresponding authors to obtain more data for the related studies that did not provide the information that our study needed. Meanwhile, we also retrieved potential records by reviewing the references in eligible studies to cover all related literatures as completely as possible.

### Principle for Study Design: PICOS

(1) Population: adults (≥18 years) except for pregnant women; (2) interventions/exposures and controls: snoring, data for which were obtained by questionnaire in the studies. If the answer was dichotomous, i.e., “yes” or “no,” the participants who reported “yes” were defined as snorers, else as non-snorers. If the answers were ranked in different frequency levels, the participants who reported “never” were defined as non-snorers, else as snorers (more details can be seen in Supplementary Table 4); (3) outcomes: metabolic syndrome and its components (hypertension/diabetes/dyslipidemia/abdominal obesity); if the participants underwent regular check-ups or reported “yes” to the question “Do you have xx (refers to a specific disease)?” or could be diagnosed after the corresponding inspection, they were defined as having the corresponding disease. The specific diagnostic criteria for each outcome are detailed in Supplementary Table 4; and (4) study designs: observational, including cross-sectional, case–control, and cohort studies.

### Inclusion and Exclusion Criteria

The inclusion criteria were as follows: (1) studies exploring the association between snoring and MetS or its components, (2) observational studies including cross-sectional, case–control, or cohort studies, and (3) ORs (95% CIs) were provided or could be calculated using relevant information.

The exclusion criteria were as follows: (1) animal studies, (2) notes, case reports, comments, guidelines, reviews, letters, conference abstracts, no full text published later, and meta-analysis, (3) not in English, (4-6) studies including children, adolescents, or pregnant participants, (7) obesity defined by body mass index (BMI) rather than abdominal obesity defined by waist circumference, (8) endpoints of type I diabetes, (9) ORs (95% CIs) were reported between different levels of snoring frequency and endpoints, but no information for the number of cases at each level was provided, and (10) duplicate publications.

### Data Extraction

Data were extracted by two authors (JM and HZ) independently. Where a disagreement occurred, a third person made the judgment. The extracted information included publication year, first author, study type, sample size, proportion of women, age, ORs (95% CIs), definition of snoring, definition of end-points, and adjusted confounders. For the ORs (95% CIs), if a study reported different values for various adjustments of covariates, the most fully adjusted ORs (95% CIs) were extracted in the final analysis. We also recorded the number of participants as well as the number of those with MetS at each level of snoring to ensure a successful dose–response meta-analysis.

### Quality Assessment

The quality assessment of the studies was performed by two authors (JM and HZ) independently. Case–control studies and cohort studies were assessed according to the Newcastle–Ottawa Scale (15), with scores ranging from 0 to 9. A score ≥6 implied higher quality, indicating that the corresponding study was credible. Cross-sectional studies were evaluated using the Agency for Healthcare Research and Quality scale (16), which includes 11 items, with “yes (1)”, “no (0)”, or “unclear (0)” categories. Scores of 0–3, 4–7, and 8–11 implied lower, medium, and higher quality, respectively.

### Statistical Analysis

In the meta-analysis, the pooled ORs (95% CIs) were used to evaluate the relationships between snoring and MetS (and its components) *via* the inverse variance method, where OR > 1 showed that snoring was a risk factor of MetS, whereas OR <1 indicated that snoring was a protective factor. If the 95% CI of the pooled OR included the null value, that is 1, no evidence was found to support the statistical association between snoring and MetS. A heterogeneity assessment was completed using the Cochrane *Q*-test and *I*^2^ index. *P* < 0.10 or *I*^2^ > 50% implied a high heterogeneity among the included studies, and a random effect model was used. Otherwise, a fixed effect model was used. Meta-regression and subgroup analysis were performed to search for the heterogeneity sources and to compare them among groups. In the sensitivity analysis, one study at a time was deleted in order to assess the influence of the deleted study on the pooled results. Begg's rank correlation test (17), Egger's linear regression test (18), and a funnel plot were used to evaluate bias among the included studies. *P* < 0.05 was considered to be statistically significant, implying the existence of bias. A symmetrical funnel plot signified that there was no bias. If there was bias, the trim and fill method was performed to assess whether the bias was caused by selective publications. After which, if the results were consistent with the previous pooled results, we consider that there was no publication bias, and the results were regarded as stable and reliable. Otherwise, an asymmetrical funnel plot indicated publication bias, and the conclusion was unreliable.

In a two-stage dose–response meta-analysis, the dose in each level of snoring frequency was defined by the median of the corresponding range. For instance, if snoring 5–7 days per week was defined as habitual snoring in the original study, the dose for habitual snoring in the dose–response meta-analysis was 6 days per week. For studies that only classified snoring frequency into “never,” “occasional,” and “regular” but no specific explanation was provided, we define their doses as “0 day/week”, “1–3 days/week”, and “4–7 days/week”, respectively. A restricted cubic spline regression model with four knots at fixed percentiles (5, 35, 65, and 95%) of the distribution was performed to evaluate the non-linear trend. A linear regression model was performed to evaluate the linear trend. The non-linear and the linear dose–response trends were tested by a chi-square test, and *p* < 0.05 signified a statistically significant non-linear or linear dose–response relationship.

## Results

### Search Results and Study Characteristics

**Figure 1:**
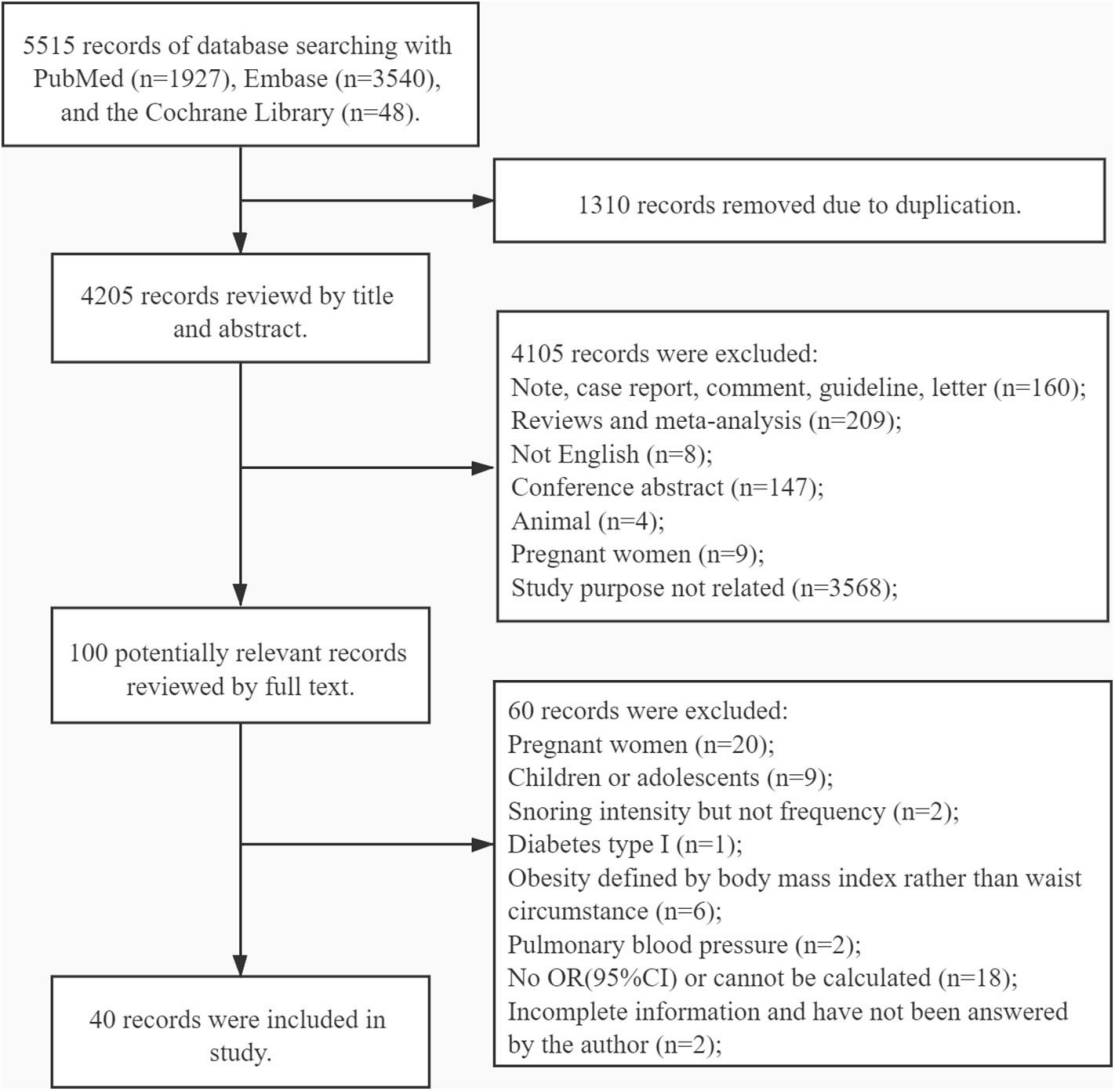
Flow chart.

A total of 5,515 potential studies were initially searched, including 1,927 records from PubMed, 3,540 from Embase, and 48 from the Cochrane Library. Ultimately, 40 studies involving 966,652 participants were reserved for the analysis after screening according to the inclusion and the exclusion criteria: 10 cohort studies, two case–control studies, and 29 cross-sectional studies. In one study, both cross-sectional and cohort study designs were used. Among these studies, 15 cross-sectional studies, two case–control studies, and seven cohort studies were classified as high-quality studies. Fourteen cross-sectional studies were classified as medium-quality studies. Three cohort studies were classified as low-quality studies (see Supplementary Tables 1–3). The studies were reported from regions spread over three continents: America (*n* = 8), Europe (*n* = 11), and Asia (*n* = 21). The process of study screening is shown in Figure 1, while the details of each study are shown in Supplementary Table 4.

### Snoring and MetS

#### Characteristics of Included Studies and Pooled OR in the Meta-Analysis

**Figure 2:**
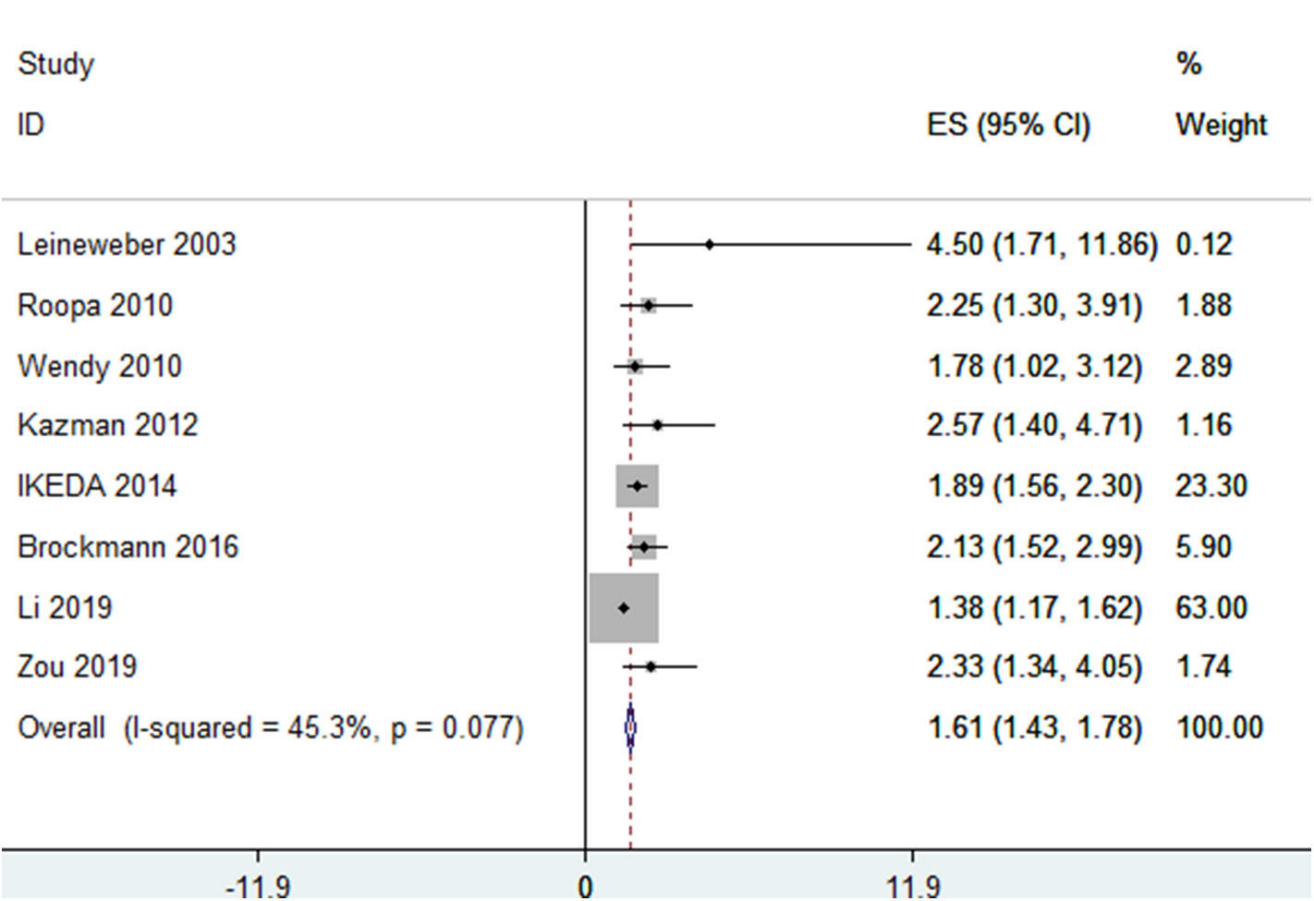
Forest plots showing the association between snoring frequency and metabolic syndrome.

In total, eight studies (8, 9, 11, 19–22), in which snoring was classified into two levels—yes/no—involving 13,065 participants reported ORs of the association between snoring and MetS. Of these eight studies, one was conducted in Europe, three in America, and four in Asia. There was heterogeneity among the included studies (*I*^2^ = 45.30%, *p* = 0.08), and the pooled OR was 1.61 (95% CI, 1.43–1.78, *p* < 0.001, random effect model) (see Figure 2).

#### Sensitivity and Subgroup Analysis

In the sensitivity analysis, when studies were deleted, one at a time, the ORs changed from 1.87 (95% CI, 1.54–2.28) to 2.05 (95% CI, 1.77–2.36), as shown in Supplementary Figure 1A. The results of the subgroup analysis and the meta-regression analysis showed statistically significant associations between snoring and MetS in different “definition of MetS (NCEP ATPIII/others)” groups (Table 1).

**Table 1:** Subgroup and meta-regression analysis for association between snoring and metabolic syndrome.

**Subgroups**		**Number**	**OR (95% CI)**	**Model**	***t*-value**	***p*-value**
Sex^*^	Female	3	1.46 (1.33-1.58)	Fixed	NA	NA
Study type	Cross sectional Cohort	7 1	1.60 (1.42–1.78) 1.78 (1.02–3.12)	Random NA	−0.29	0.78
Region	Asian Others	4 4	1.55 (1.36–1.74) 2.11 (1.55–2.67)	Random Fixed	−1.31	0.24
Quality	High Median or low	6 2	1.58 (1.40–1.76) 2.37 (1.35–3.40)	Random Fixed	−0.95	0.38
Definitions of MetS	NCEP ATPIII	8	1.67 (1.57–1.74)	Random	6.52	<0.001
	Others	4	1.54 (1.35–1.73)	Random		
Adjustment for confounders smoke	Yes No	5 3	1.56 (1.37–1.74) 2.42 (1.58–2.91)	Random Fixed	−1.58	0.164
Adjustment for confounders alcohol	Yes No	5 3	1.56 (1.37–1.74) 2.42 (1.58–2.91)	Random Fixed	−1.58	0.164
Adjustment for confounders BMI	Yes No	2 6	1.44 (1.23–1.66) 1.96 (1.64–2.28)	Random Fixed	−1.50	0.18
Adjustment for confounders physical activity	Yes No	4 4	1.54 (1.35–1.73) 2.26 (1.66–2.86)	Random Fixed	−1.93	0.10
Adjustment for confounders emotion	Yes No	2 6	1.88 (1.53–2.23) 1.51 (1.30–1.72)	Fixed Fixed	−0.39	0.71
Adjustment for confounders sleep	Yes No	1 7	2.25 (1.52–2.99) 1.57 (1.39–1.76)	NA Fixed	0.32	0.68

* *The studies reported ORs (95% CIs) for female only, but no for male*.

#### Evaluation of Publication Bias

**Figure 3:**
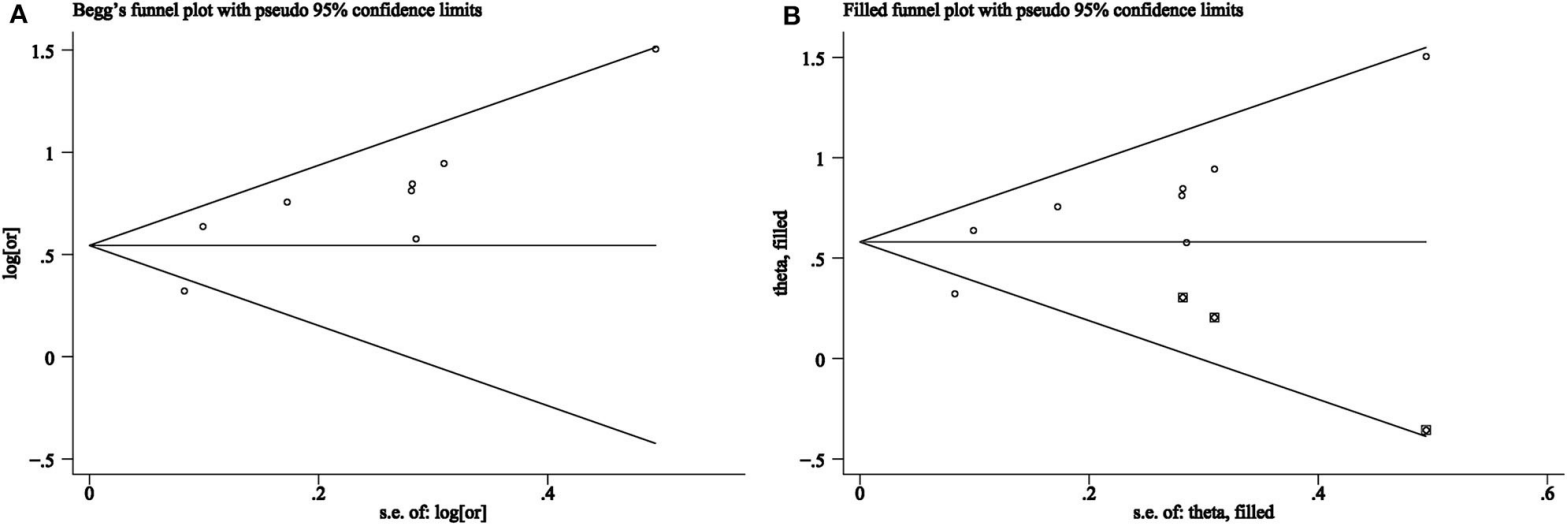
**(A)** Funnel plots of association between snoring frequency and metabolic syndrome. **(B)** Trim and fill analysis for an asymmetric funnel plot.

Although the *p*-value for Egger's test was 0.02, implying bias among included studies, the *p*-value for Begg's test was 0.17, implying no bias. The funnel plot can be seen in Figure 3A. After evaluation using the trim and fill method, the OR was 1.79 (95% CI, 1.48–2.17, in the random effect model), which is consistent with previous results, implying the absence of publication bias. Furthermore, the funnel plot in the trim and fill analysis was symmetrical, as shown in Figure 3B.

#### Characteristics of the Included Studies and Results in the Dose–Response Meta-Analysis

**Figure 4:**
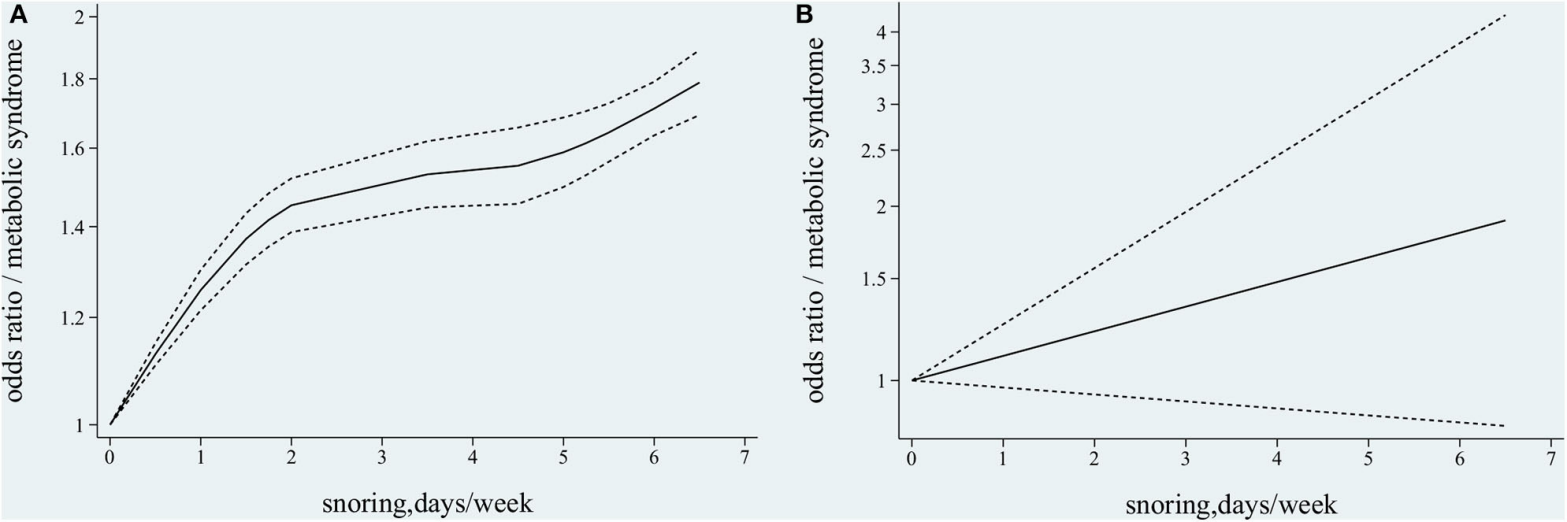
Association between snoring frequency and metabolic syndrome (MetS) by dose–response meta-analysis. **(A)** Non-linear trend between snoring frequency and MetS. **(B)** Linear trend between snoring frequency and MetS.

The six studies from five publications (8, 23–26) with 91,346 participants reported associations between different snoring frequencies and MetS; only one study (26) reported separately on the data of men and women. The participants were all Asian, except those reported by Sabanayagam et al. (24). The *Q*-test result indicated the presence of heterogeneity (χ^2^ = 96.54, *p* < 0.001) among the six studies. Following the use of the non-linear and the linear random effect dose–response models, the hypothesis testing for a non-linear trend was found to be statistically significant (χ^2^ = 75.01, *p* < 0.001, Figure 4A), but not significant for a linear trend (χ^2^ = 2.31, *p* = 0.13, Figure 4B). The monotonically increasing relationship found between snoring frequency and MetS was shown with ORs of 1.13 (95% CI, 1.11–1.15) for 0.5 day/week, 1.26 (95% CI, 1.21–1.30) for 1 day/week, 1.37 (95% CI, 1.31–1.43) for 1.5 days/week, 1.45 (95% CI, 1.39–1.52) for 2 days/week, 1.53 (95% CI, 1.45–1.62) for 3.5 days/week, 1.55 (95% CI, 1.45–1.66) for 4.5 days/week, 1.59 (95% CI, 1.50–1.68) for 5 days/week, 1.64 (95% CI, 1.56–1.73) for 5.5 days/week, 1.71 (95% CI, 1.63–1.79) for 6 days/week, and 1.79 (95% CI, 1.69–1.89) for 6.5 days/week, as shown in Figure 4A.

### Snoring and Hypertension

#### Characteristics of Studies and Pooled OR in the Meta-Analysis

**Figure 5:**
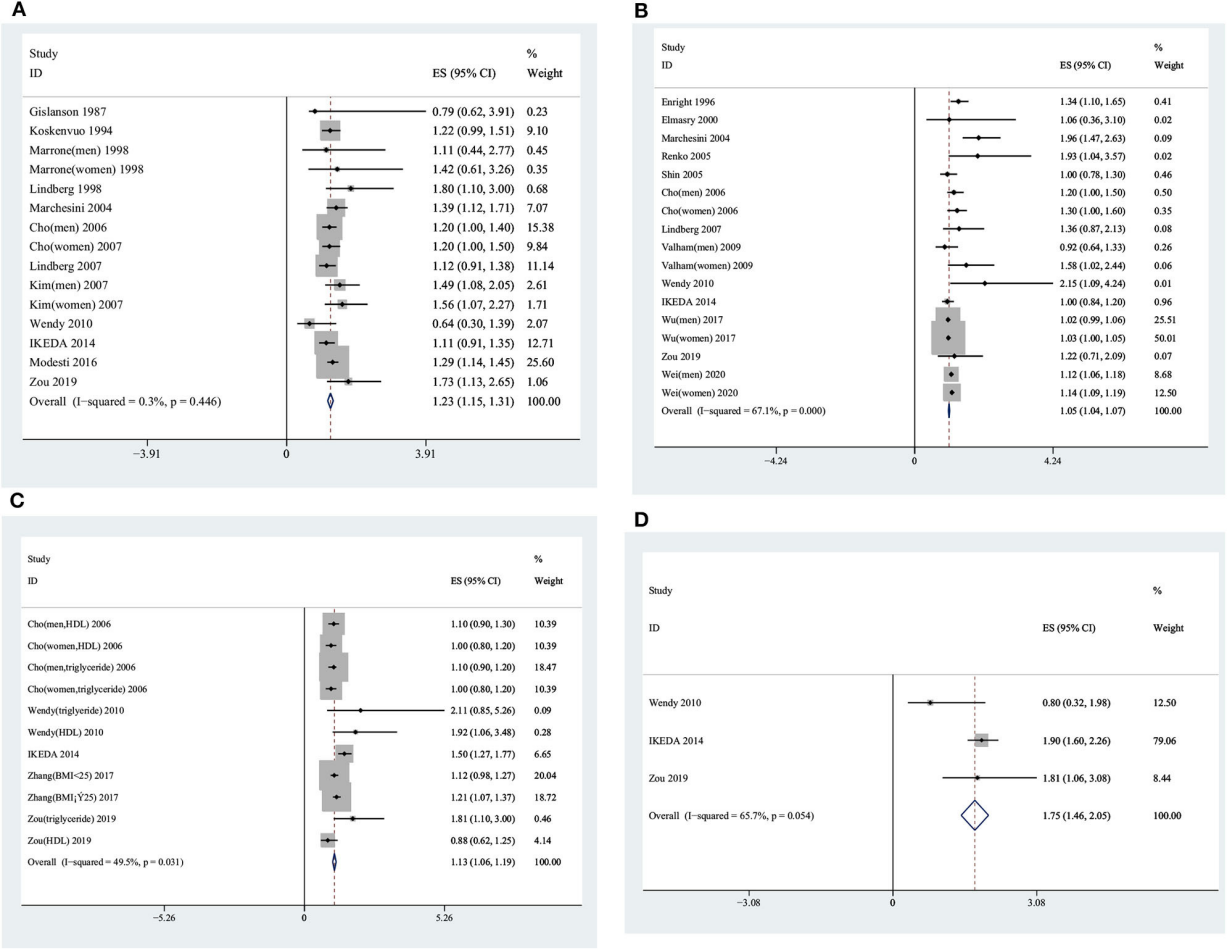
Forest plots showing the association between snoring frequency and the components of metabolic syndrome. **(A)** Snoring frequency and blood pressure. **(B)** Snoring frequency and glucometabolism. **(C)** Snoring frequency and lipid metabolism. **(D)** Snoring frequency and abdominal obesity.

Of the 15 studies from 12 publications (6, 10, 11, 22, 27–34) with 41,463 participants that reported ORs (95% CIs) of snoring and hypertension, one study used a case–control design, three studies used a cohort design, and eight studies used a cross-sectional design. Three studies (30, 32, 33) provided information on men and women separately. There was no heterogeneity among the 15 studies (*I*^2^ = 0.30%, *p* = 0.45); thus, the fixed effect model was used for further analysis. The pooled OR was 1.23 (95% CI, 1.15–1.31, fixed effect model; see Figure 5).

#### Sensitivity and Subgroup Analysis

The sensitivity analysis showed stable results, with pooled ORs ranging from 1.24 (95% CI, 1.15–1.34) to 1.27 (95% CI, 1.19–1.36), as shown in Supplementary Figure 1B. In men and women, the pooled ORs were 1.24 (95% CI, 1.10–1.39) and 1.19 (95% CI, 1.03–1.36), respectively. Furthermore, for Asians and other ethnicities, the pooled ORs were 1.22 (95% CI, 1.10–1.34) and 1.22 (95% CI, 1.13–1.34), respectively (see Supplementary Table 5).

#### Evaluation of Publication Bias

There was no publication bias among the included studies (*p* = 0.37 for Begg's test and *p* = 0.87 for Egger's test), and the funnel plot was symmetrical (see Supplementary Figure 2A).

#### Characteristics of Studies and Results in the Dose–Response Meta-Analysis

Of a total of 13 studies from eight articles (8, 25, 26, 35–41) with 589,658 participants included in the dose–response meta-analysis, three (26, 35, 40) reported ORs (95% CIs) on the association between snoring and hypertension in men and women separately. The participants in all studies were Asians, except for one (35) in which they were American. There was heterogeneity among the 13 dose–response studies (χ^2^ = 264.59, *p* < 0.001); therefore, the random effect model was used. Both non-linear and linear dose–response models showed statistically significant results (non-linear, χ^2^ = 126.42, *p* < 0.001; linear, χ^2^ = 5.22, *p* = 0.02). The non-linear curve showed ORs of 1.07 (95% CI, 1.06–1.09) for 0.5 day/week, 1.21 (95% CI, 1.15–1.27) for 1.5 days/week, 1.26 (95% CI, 1.18–1.34) for 2 days/week, 1.31 (95% CI, 1.20–1.44) for 3 days/week, 1.32 (95% CI, 1.19–1.47) for 3.5 days/week, 1.34 (95% CI, 1.17–1.53) for 4.5 days/week, 1.36 (95% CI, 1.17–1.58) for 5 days/week, 1.38 (95% CI, 1.17–1.63) for 5.5 days/week, 1.44 (95% CI, 1.18–1.75) for 6.5 days/week, and 1.47 (95% CI, 1.18–1.82) for 7 days/week (see Supplementary Figure 3A). The linear model indicated an increased risk of hypertension of 1.07 (95% CI, 1.01–1.13) times for each additional day of snoring per week (see Supplementary Figure 3B).

### Snoring and Glycometabolism

#### Characteristics of Studies and Pooled OR in the Meta-Analysis

Of a total of 17 studies from 13 articles (7, 10, 11, 22, 31, 32, 34, 42–47) with 584,912 participants reporting the ORs (95% CIs) between snoring and glycometabolism, four (32, 45–47) studies provided information on men and women separately. The pooled OR was 1.05 (95% CI, 1.04–1.07, random effect model) with significant heterogeneity (*I*^2^ = 67.10%, *p* < 0.01) (see Figure 5).

#### Sensitivity and Subgroup Analysis

The sensitivity analysis showed stable pooled results ranging from 1.05 (95% CI, 1.04–1.07) to 1.10 (95% CI, 1.08–1.13), as shown in Supplementary Figure 1C. The subgroup analysis showed pooled ORs of 1.05 (95% CI, 1.02–1.08) in men and 1.05 (95% CI, 1.03–1.07) in women. Similar significant results were derived in different regions, with ORs of 1.05 (95% CI, 1.03–1.07) for Asians and 1.32 (95% CI, 1.14–1.50) for other ethnicities. For different study types, the pooled ORs were 1.03 (95% CI, 1.01–1.05) in cross-sectional studies and 1.13 (95% CI, 1.10–1.71) in cohort studies (see Supplementary Table 6).

#### Evaluation of Publication Bias

The *p*-value for Egger's linear test was 0.01, implying the existence of bias among the studies. However, the *p*-value for Begg's rank was 0.56, indicating no bias. The funnel plot can be seen in Supplementary Figure 2B. Trim and fill analysis showed a pooled OR of 1.10 (95% CI, 1.03–1.16), and a symmetrical funnel plot was found, implying no publication bias (Supplementary Figure 2C).

#### Characteristics of Studies and Results in the Dose–Response Meta-Analysis

In 14 studies, there were 11 articles (8, 25, 26, 36, 37, 40, 46, 48–51) with 299,423 participants in the dose–response meta-analysis, and three (26, 40, 46) studies reported a relationship between snoring and glycometabolism in men and women separately. There was heterogeneity among the 14 dose–response studies (χ^2^ = 155.56, *p* < 0.001); thus, a random effect model was performed. Both the non-linear and the linear dose–response models were statistically significant (non-linear, χ^2^ = 7.60, *p* = 0.02; linear, χ^2^ = 12.16, *p* = 0.005). The non-linear model displayed ORs of 1.04 (95% CI, 1.02–1.06) for 0.5 day/week, 1.12 (95% CI, 1.06–1.19) for 1.5 days/week, 1.15 (95% CI, 1.08–1.23) for 2 days/week, 1.21 (95% CI, 1.09–1.34) for 3.5 days/week, 1.25 (95% CI, 1.09–1.43) for 4.5 days/week, 1.28 (95% CI, 1.10–1.48) for 5 days/week, 1.32 (95% CI, 1.12–1.55) for 5.5 days/week, 1.36 (95% CI, 1.14–1.62) for 6 days/week, 1.40 (95% CI, 1.16–1.71) for 6.5 days/week, and 1.45 (95% CI, 1.18–1.79) for 7 days/week (see Supplementary Figure 3C). The linear model indicated an increased risk of hypertension of 1.05 (95% CI, 1.02–1.08) times for each additional day of snoring per week (see Supplementary Figure 3D).

### Snoring and Lipid Metabolism

#### Characteristics of Studies and OR in the Meta-Analysis

In 11 studies, five articles (10, 11, 22, 32, 52) with 25,300 participants provided information on the association between snoring and lipid metabolism. The pooled OR was 1.13 (95% CI, 1.06–1.19, random effect model), with significant heterogeneity (*I*^2^ = 49.5%, *p* = 0.03) (see Figure 5).

#### Sensitivity and Subgroup Analysis

As shown in Supplementary Figure 1D, the pooled ORs ranged from 1.12 (95% CI, 1.02–1.22) to 1.19 (95% CI, 1.07–1.33) by deleting each one of the included studies. According to the subgroup analysis of different lipid indexes, we found a pooled OR of 1.09 (95% CI, 1.00–1.18) for low-HDL and 1.08 (95% CI, 1.00–1.17) for triglyceride (see Supplementary Table 7).

#### Evaluation of Publication Bias

The *p*-values were 0.94 and 0.41 for Begg's test and Egger's tests, respectively; thus, the funnel plot was symmetrical as shown in Supplementary Figure 2D.

#### Characteristics of Studies and Results in the Dose–Response Meta-Analysis

Of seven studies from three articles (8, 26, 37), 81,469 participants were included in the dose–response meta-analysis. Huang et al. (37) reported an association between snoring and dyslipidemia (abnormal triglyceride or HDL); Li et al. (8) reported relationships between snoring and high-triglyceride and low-HDL separately, while Kim et al. (26) reported a relationship between snoring and triglyceride and HDL in men and women separately. The χ^2^ value for heterogeneity was 106.48 (*p* < 0.001); thus, a random effect model was used. There were non-linear and linear dose–response trends of the association between snoring and dyslipidemia (non-linear, χ^2^ = 29.68, *p* < 0.001; linear, χ^2^ = 4.86, *p* = 0.03; random effect model). The non-linear model showed ORs of 1.21 (95% CI, 1.11–1.32) for 1.5 days/week, 1.26 (95% CI, 1.13–1.41) for 2 days/week, 1.42 (95% CI, 1.11–1.81) for 4.5 days/week, 1.46 (95% CI, 1.11–1.91) for 5 days/week, and 1.60 (95% CI, 1.13–1.27) for 6.5 days/week (see Supplementary Figure 4A).The linear model indicated an increased risk of dyslipidemia of 1.08 (95% CI, 1.01–1.15) times for each additional day of snoring per week (see Supplementary Figure 4B).

Subgroups were derived according to different lipid parameters for further analysis. The results of the *Q*-test indicated the presence of heterogeneity (χ^2^ = 1.10, *p* = 0.95) among “studies reporting on HDL,” implying that a fixed effect model was needed. There were linear trends but no non-linear trend between snoring and HDL (non-linear, χ^2^ = 0.68, *p* = 0.71, Supplementary Figure 4C; linear, χ^2^ = 49.65, *p* < 0.001, Supplementary Figure 4D; fixed effect model). The linear model indicated an increased risk of abnormal HDL of 1.03 (95% CI, 1.02–1.04) times for each additional day of snoring per week (see Supplementary Figure 4D).

The results of the *Q*-test indicated the presence of heterogeneity (χ^2^ = 20.29, *p* = 0.001) among “studies reporting on triglyceride,” implying that a random effect model was needed. There were non-linear but no linear dose–response relationships between snoring frequency and triglyceride (non-linear, χ^2^ = 49.21, *p* < 0.001, Supplementary Figure 4E; linear, χ^2^ = 0.66, *p* = 0.42, Supplementary Figure 4F; random effect model). The monotonous curve showed ORs of 1.25 (95% CI, 1.17–1.33) for 1.5 days/week, 1.30 (95% CI, 1.21–1.41) for 2 days/week, 1.37 (95% CI, 1.18–1.59) for 4.5 days/week, 1.37 (95% CI, 1.17–1.61) for 5 days/week, and 1.42 (95% CI, 1.15–1.74) for 6.5 days/week (see Supplementary Figure 4E).

### Snoring and Abdominal Obesity

#### Characteristics of Studies and Pooled OR in the Meta-Analysis

There was heterogeneity among four studies (10, 11, 22) (*I*^2^ = 65.70%, *p* = 0.05), with 5,614 participants. The pooled OR (95% CI) was 1.75 (95% CI, 1.46–2.05, random effect model), as shown in Figure 5.

#### Sensitivity Analysis

The sensitivity analysis showed that the ORs ranged from 1.47 (95% CI, 0.93–2.33) to 1.89 (95% CI, 1.60–2.23) by deleting the included studies one at a time (Supplementary Figure 1E).

#### Evaluation of Publication Bias

The *p*-values for Egger's linear test and Begg's rank test were 0.296 and 0.387, respectively, which indicated that there was no publication bias and a symmetrical funnel plot was found (see Supplementary Figure 2E).

#### Characteristics of Studies and Results in the Dose–Response Meta-Analysis

In three studies from two articles (8, 26) with 77,183 participants, information on the dose–response association between snoring and abdominal obesity was reported. Kim et al. (26) reported on men and women separately. The χ^2^ for heterogeneity was 5.27 (*p* = 0.38); hence, a fixed effect model was used. There were non-linear and linear dose–response trends in the relationship between snoring and abdominal obesity (non-linear, χ^2^ = 144.92, *p* < 0.001; linear, χ^2^ = 1,591.51, *p* < 0.001; fixed effect model). The monotonous curvilinear association showed ORs of 1.63 (95% CI, 1.56–1.70) for 1.5 days/week, 1.77 (95% CI, 1.69–1.85) for 2 days/week, 1.95 (95% CI, 1.81–2.09) for 4.5 days/week, 2.06 (95% CI, 1.93–2.20) for 5 days/week, and 2.74 (95% CI, 2.50–2.89) for 6.5 days/week, as shown in Supplementary Figure 3E. The linear model indicated an increased risk of hypertension of 1.17 (95% CI, 1.16–2.89) times for each additional day of snoring per week, as shown in Supplementary Figure 3F.

## Discussion

This meta-analysis of one cohort study and seven cross-sectional studies displayed a statistically significant linkage between snoring and MetS. Furthermore, the dose–response meta-analysis of six other studies revealed a non-linear trend of the role of snoring on MetS. Our previous study revealed a similar association even when the interaction effect between snoring and obesity was considered on the prevalence of MetS (8). The study of Zou et al. showed an association between snoring and MetS after excluding obstructive sleep apnea (OSA) (22). In addition, their studies provided evidence that the relationship was more obvious in men than in women. The present study provided pooled ORs for women and the entire population (but not in men because of the limited number of studies) of 1.46 (95% CI, 1.33–1.58) and 1.61 (95% CI, 1.43–1.78), respectively. However, we found a more remarkable relationship in the entire population than in women alone. Thus, we made a bold guess that the association between snoring and MetS would probably be significant in men and may be more pronounced than in women, as mentioned above. Such an assumption requires confirmation in future studies. Moreover, we observed that the biomechanisms explaining the relationship between snoring and MetS, which were not clearly clarified until now, involve two major pathways partly explaining for the linkage: first, mechanical injury: mechanical damage to the endothelial wall and inflammatory cascade caused by snoring may aggravate carotid atherosclerotic plaque, subsequently contributing to MetS (53, 54); second is the disorder of the neuroendocrine system: microarousals and intermittent hypoxia caused by snoring during sleep have a direct adverse impact on sleep quality (19), thereby increasing the excitability of the sympathetic nervous system and influencing the hypothalamic–pituitary–adrenal axis, which may contribute to metabolic disturbance (55, 56).

This study showed that snoring was a risk factor for hypertension both in pooled results and in the dose–response meta-analysis. In the subgroup analysis of different study designs, case–control studies showed the opposite results of a non-significant OR of 1.25 (95% CI, 0.37–2.12). The two case–control studies were both by Marrone et al. (30), for both men and women with ORs of 1.11 (95% CI, 0.44–2.77) and 1.42 (95% CI, 0.61–3.26), respectively. Moreover, the sample size was small, i.e., including only 45 pairs. Therefore, the non-significant conclusions may be due to the wider confidence interval resulting from the small number of participants. Meanwhile, in the non-linear dose–response meta-analysis (Supplementary Figure 3A), an increased risk of hypertension occurred initially, but later the upward trend slows down with increasing snoring frequency. This might have occurred because of the hazard ratio (HR) between snoring frequency and hypertension that increased with age in women but decreased in men, as reported by previous studies (38). In the study of Lee et al. (38), the average age and the proportion of men in the “habitual” snorers group were higher than those in either the “never” or the “occasional” snorers group, which explained why the HR for “habitual” snorers was lower than that for the “occasional” snorers group, with the “never” snorers group as reference. This phenomenon was also reported by Kim et al. (26). Secondly, our non-linear analysis included the studies of Lee et al. (38) and Kim et al. (26), and both reported lower effect value in habitual snorers than in the “occasional” group. In addition, both studies accounted for 34.64% (77,839/224,745) of the total sample size in the dose–response analysis, implying that these two studies had a larger weight and would have a greater influence on the final results. The biomechanisms underlying the relationship between cardiovascular issues and snoring have been partly clarified by results from elevated circulating catecholamine owing to an excited sympathetic nervous system (48), increased vasoconstrictor sensitivity as a result of vascular remodeling due to the nocturnal blood pressure surges (57), the development of atherosclerosis as a consequence of endothelial wall damage, and the inflammatory response caused by snoring vibration (53).

As mentioned in the introduction, Xiong et al. performed a meta-analysis on the relationship between snoring and diabetes in 2016 (12), but only eight studies were included. In our analysis, we updated the review and performed both typical and dose–response meta-analysis by adding 17 articles. Our results showed that the association between snoring and glycometabolism displayed in typical and dose–response meta-analysis was significant. All the subgroup analysis results stratified by sex, study type, regions, and quality of studies showed statistically significant associations between glycometabolism and snoring frequency. In terms of mechanisms of the association between snoring frequency and diabetes, one study found that snoring was related to increasing urinary albumin level, which is a risk factor for diabetes (58). Moreover, snoring and the accompanying temporary cessation of breathing have been found to excite the sympathetic nervous system (59). Another study showed increased oxidative stress as a consequence of snoring, which, in turn, increases cholamine and cortisol levels (48), thereby impairing glucose homeostasis and increasing glycogenesis and gluconeogenesis, ultimately leading to insulin resistance (44, 60).

For an association between snoring and dyslipidemia, Cho et al. (32) showed that snorers had higher prevalence rates of hyper-total cholesterol and lower-HDL, but the association between the two disappeared after adjusting for various confounders. However, Zhang et al. (52) concluded that a significant relationship exists between snoring and dyslipidemia in those with a BMI <25 kg/m^3^, but not in those with a BMI ≤ 25kg/m^3^ in a multivariable analysis. Furthermore, Huang et al. (37) concluded that a strong correlation was found between snoring and MetS' components, especially dyslipidemia. In our study, both typical and dose–response meta-analysis displayed significant conclusions for the relationship between snoring and dyslipidemia. The subgroup analysis results for different lipid indices, HDL and triglyceride, were also positive, with less heterogeneity (*I*^2^ = 4.4% for HDL; *I*^2^ = 0.0% for triglyceride).

The present study showed a significant association between snoring and abdominal obesity in typical and dose–response meta-analysis. Previous studies have reported obesity as a major cause of snoring (61), whereas snoring can in turn accelerate the development of central obesity. Perry et al. (62) concluded that abdominal obesity was not only a component of MetS but was also related to insulin resistance, which is another of MetS' component. Another study showed an interaction effect of obesity between snoring and MetS (8). Zhang et al. (52) also concluded that snoring was related to HDL and cholesterol in participants with a BMI ≥ 25 kg/m^2^, but not in participants with a BMI <25 kg/m^2^. Similar studies (9, 43) also showed evidence that snoring was a risk factor of metabolic disorders, but the association weakens and even disappears when considering obesity-related indicators as confounders, such as BMI or waist-to-hip ratio. Moreover, another previous study showed that the interaction between obesity and snoring on metabolic disorders may be caused by activating the chronic inflammatory response and/or adipocyte factor disturbance (23). Therefore, all these studies showed a complex association between snoring, obesity, and MetS, pointing the direction for future study.

Between-study heterogeneity is common in meta-analysis. The meta-regression results showed that the different definition criteria for MetS are associated with heterogeneity in the meta-analysis for MetS (Table 1). The study also showed that regions, adjustment for smoking, alcohol consumption, and physical activity are associated with heterogeneity in the meta-analysis for glycometabolism (Supplementary Table 6). The adjustment for smoking, alcohol consumption, physical activity, and emotion was associated with heterogeneity in the meta-analysis for dyslipidemia (Supplementary Table 7). Therefore, these factors should be considered on the topic of association between snoring and glycometabolism or dyslipidemia in the future.

To the best of our knowledge, this is the first comprehensive and systematic review to reveal the associations between snoring frequency and MetS and its components. Meanwhile, our analysis provides conclusion on the dose–response trend between snoring and MetS, which provided support for further research on the causal association between snoring frequency and MetS. However, several limitations should be noted. Firstly, the risk factors for MetS still remain unclear; therefore, the confounding variables in various studies differed. However, we extracted the ORs with most adjusted factors in our analysis. Secondly, most studies included in our analysis were cross-sectional or case–control studies that did not incorporate time sequence criteria for determining causality. Furthermore, only one study (22) excluded the participants of OSA and reported the effect value between simple snoring and MetS. Snoring is a precursor to OSA, and the relationship between snoring and MetS found in our study may be mediated by OSA. This means that it is unclear whether simple snoring, meaning an absence of OSA, would be related to MetS and its components.

In conclusion, we observed significant associations between snoring frequency and MetS and its components both in typical and dose–response meta-analyses. Our findings, therefore, have implications for metabolic dysfunction management. Early detection of snoring and early intervention in snorers may decrease the risk of MetS. Because of the intrinsic limitations of the included studies, more prospective cohort studies with participants who are simple snorers, thus excluding patients with OSA, are still needed to establish the potential causal relationship between snoring and MetS.

## Author Contributions

TW was responsible for the study concept and design. JM and HZ completed the study selection and study evaluation. HW helped to perform the dose–response meta-analysis. JM performed the information extraction and wrote the manuscript. QG, HS, SH, LM, and TW reviewed and edited the manuscript. All authors read and approved the final manuscript.

## Conflict of Interest

The authors declare that the research was conducted in the absence of any commercial or financial relationships that could be construed as a potential conflict of interest.

## Abbreviations

AHRQ: agency for healthcare research and quality
BMI: body mass index
CI: confidence interval
HDL: high-density lipoprotein
MetS: metabolic syndrome
MeSH: medical subject heading
MOOSE: Meta-analysis of Observational Studies in Epidemiology group
OR: odds ratio
OSA: obstructive sleep apnea
PRISMA: Preferred Reporting Items for Systematic Reviews and Meta-analysis.
