# Supplementary figures and images for: Association Between Self-Reported Snoring and Metabolic Syndrome: A Systematic Review and Meta-Analysis
Source: Front Neurol. 2020 Oct 2;11:517120. doi: 10.3389/fneur.2020.517120 (PMC7566901; doi:10.3389/fneur.2020.517120)

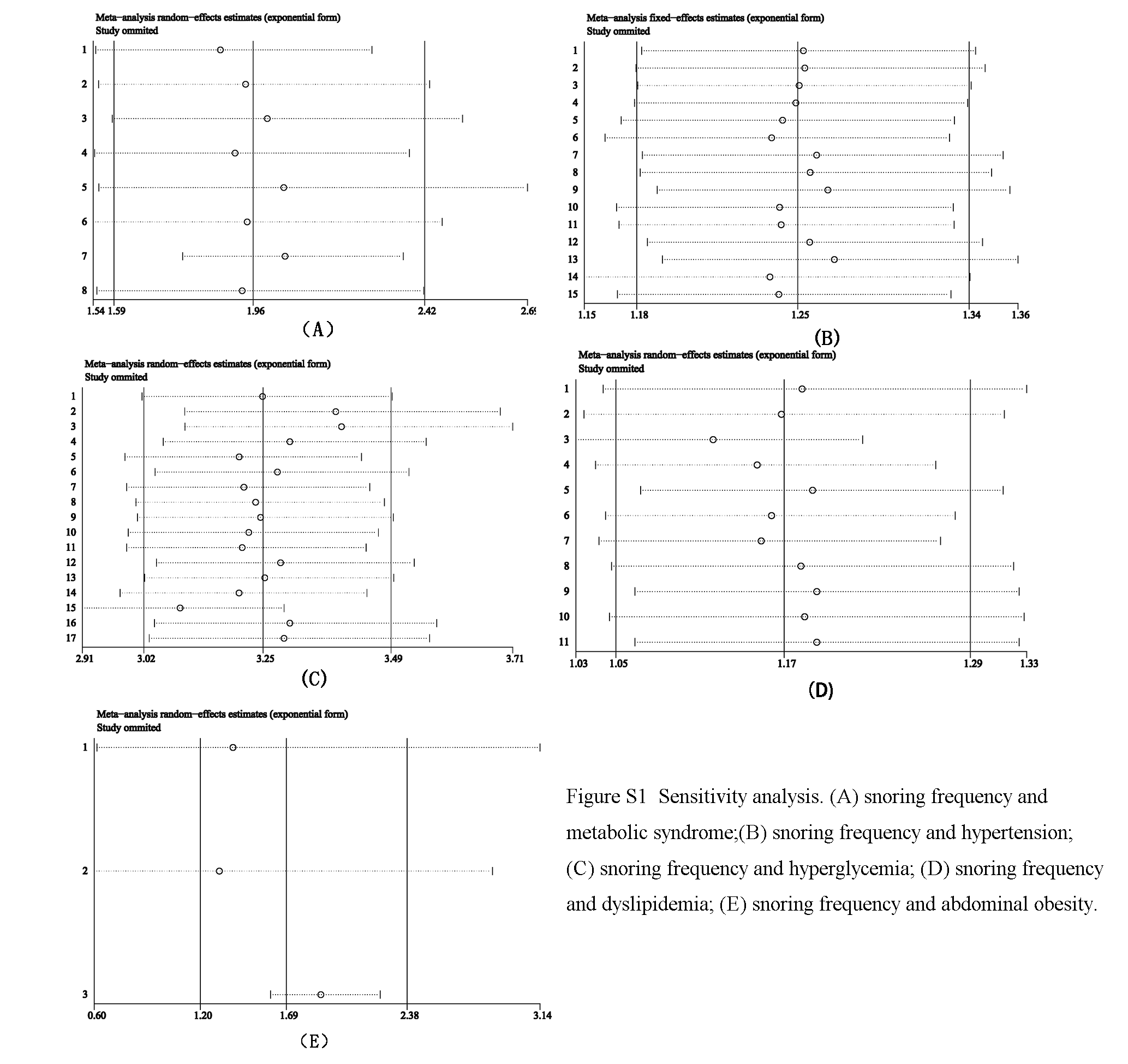

Supplement: Supplementary file 1 [file Image_1.TIF]

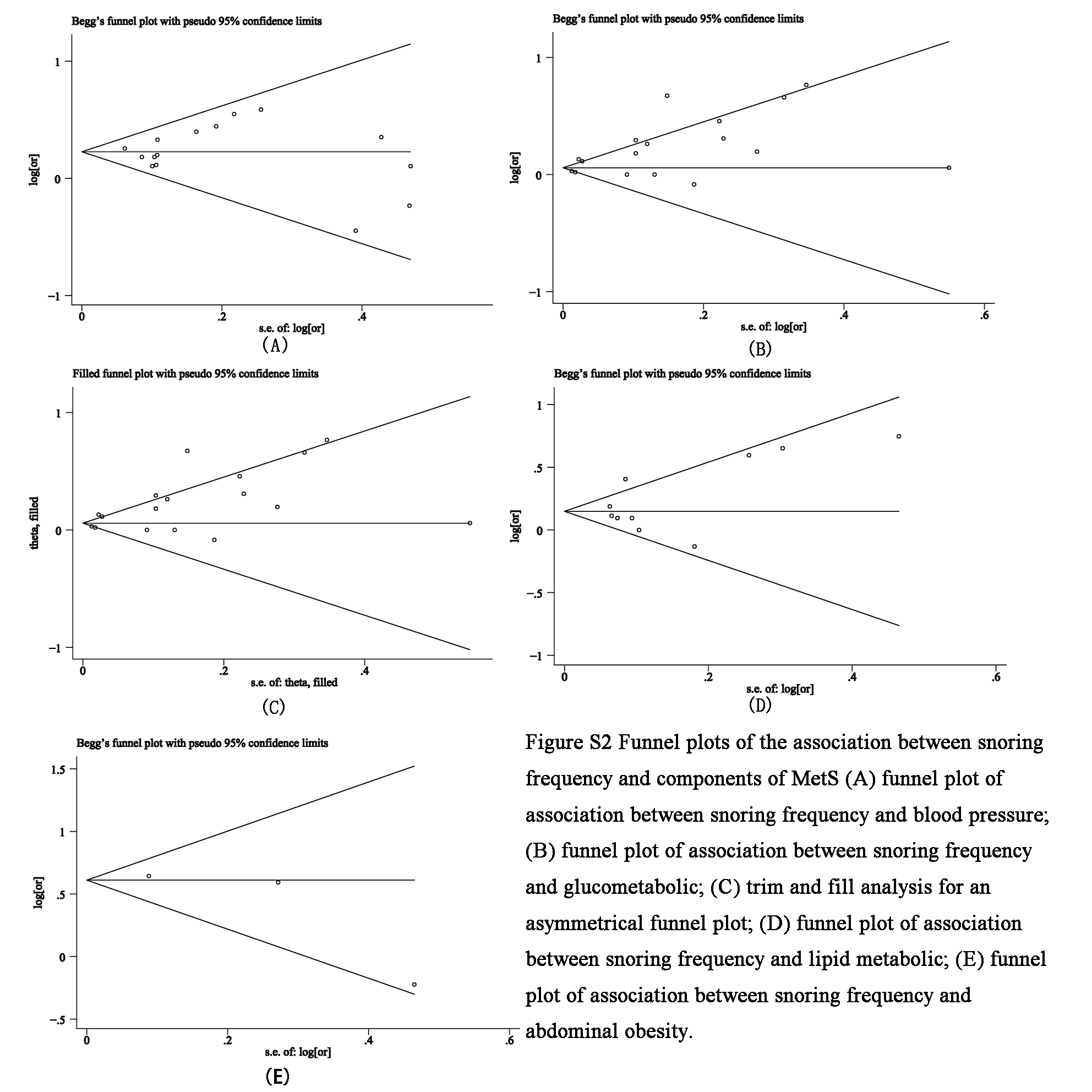

Supplement: Supplementary file 2 [file Image_2.TIF]

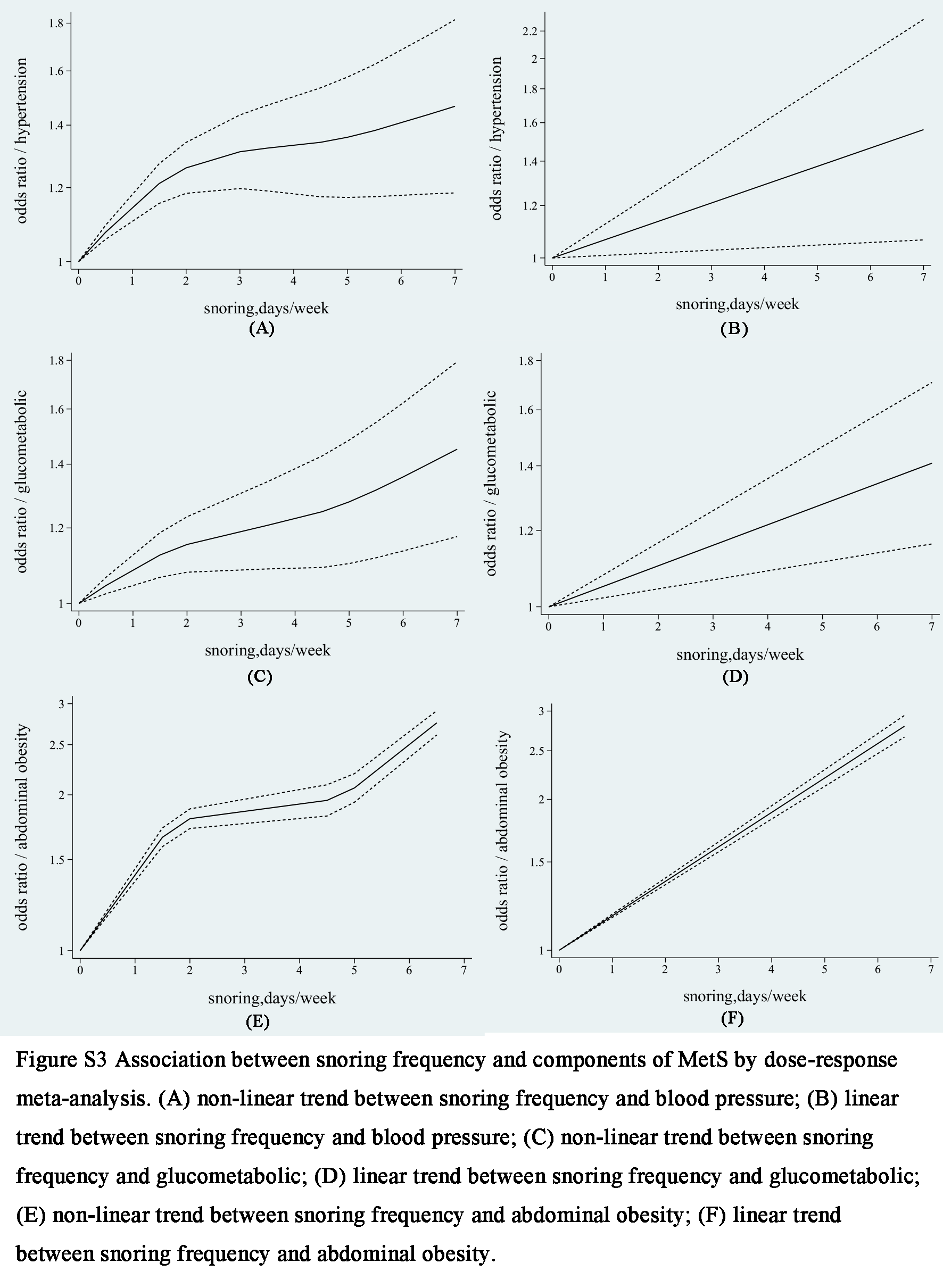

Supplement: Supplementary file 3 [file Image_3.TIF]

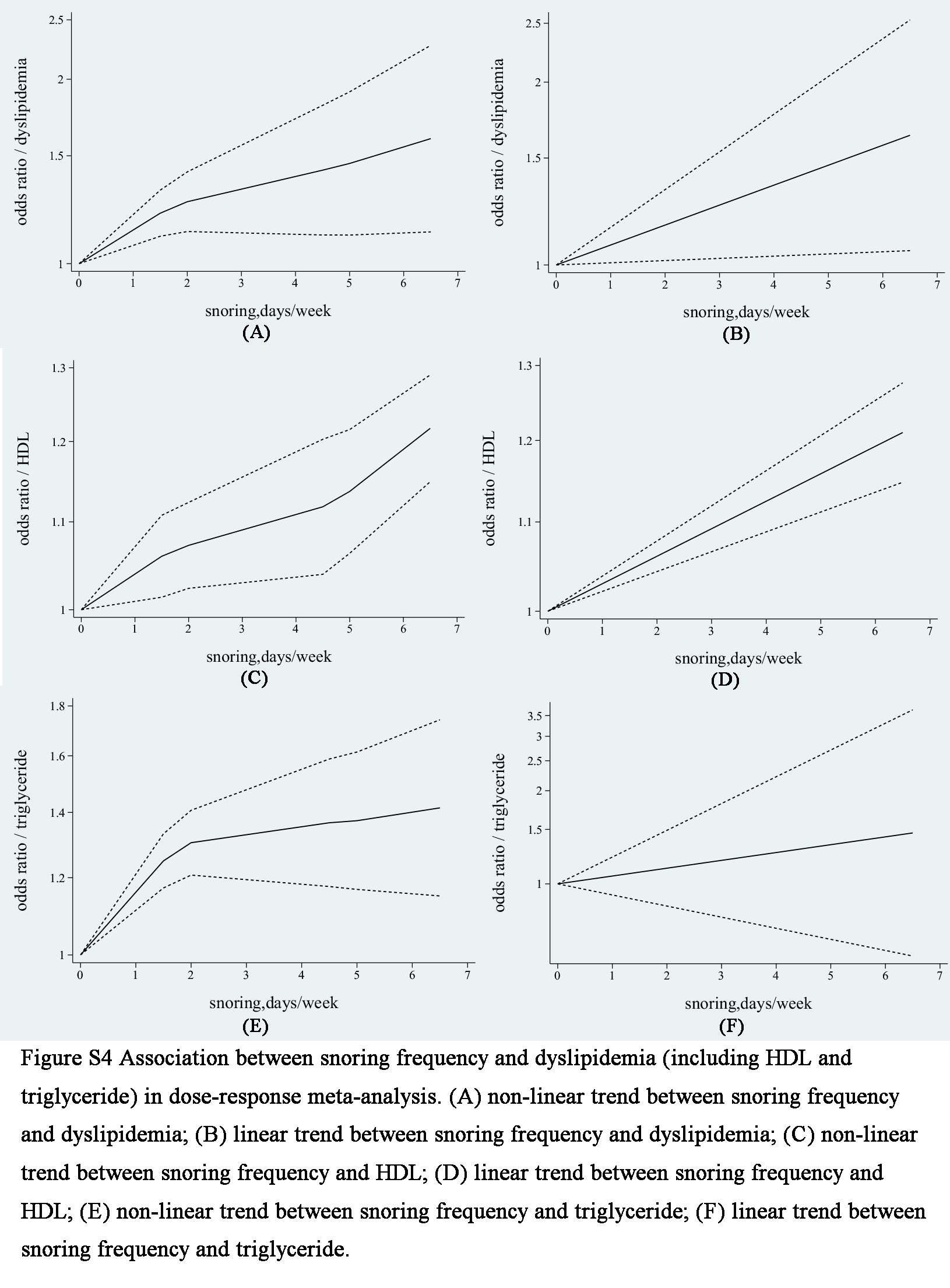

Supplement: Supplementary file 4 [file Image_4.TIF]
