# Supplementary material for: Association Between Self-Reported Snoring and Metabolic Syndrome: A Systematic Review and Meta-Analysis
Source: Front Neurol. 2020 Oct 2;11:517120. doi: 10.3389/fneur.2020.517120 (PMC7566901; doi:10.3389/fneur.2020.517120)
Supplement: Supplementary file 5 [file Table_1.doc]

Table S1 Quality Assessment for Cross-sectional Studies

| Study  Item | Gislanson  1987 | Koskenvuo  1994 | Enright  1996 | Hu  1999 | Leineweber  2003 | Marchesini  2004 | Shin  2005 | Cho  2006 | Lindberg  2007 |
| --- | --- | --- | --- | --- | --- | --- | --- | --- | --- |
| (1) Define the source of information (survey, record review).(1) | yes | yes | yes | yes | yes | yes | yes | yes | yes |
| (2) List inclusion and exclusion criteria for exposed and unexposed subjects (cases and controls) or refer to previous publications.(1) | yes | yes | yes | yes | yes | yes | yes | yes | yes |
| (3) Indicate time period used for identifying patients.(1) | yes | no | yes | yes | no | no | yes | no | yes |
| (4) Indicate whether or not subjects were consecutive if not population-based.(1) | yes | yes | yes | yes | yes | yes | yes | yes | yes |
| (5) Indicate if evaluators of subjective components of study were masked to other aspects of the status of the participants.(1) | unclear | unclear | unclear | unclear | unclear | unclear | unclear | unclear | unclear |
| (6) Describe any assessments undertaken for quality assurance purposes (e.g., test/retest of primary outcome measurements).(1) | no | no | yes | no | yes | yes | yes | yes | no |
| (7) Explain any patient exclusions from analysis.(1) | no | no | yes | yes | yes | no | no | no | no |
| (8) Describe how confounding was assessed and/or controlled.(1) | unclear | yes | yes | yes | yes | yes | yes | yes | yes |
| (9) If applicable, explain how missing data were handled in the analysis.(1) | no | no | no | no | no | no | no | no | no |
| (10) Summarize patient response rates and completeness of data collection.(1) | yes | yes | yes | no | yes | no | no | yes | yes |
| (11) Clarify what follow-up, if any, was expected and the percentage of patients for which incomplete data or follow-up was obtained.(1) | yes | no | yes | no | yes | yes | no | no | yes |
| Total (11) | 6 | 5 | 9 | 6 | 8 | 6 | 6 | 6 | 7 |

Table S1 Quality Assessment for Cross-sectional Studies (continued)

| Study  Item | Valham  2009 | Roopa  2010 | Sabanayagam  2011 | Sabanayagam  2012 | Kazman  2012 | Ikeda  2014 | Wang 2015 | Brockman  2016 | Modesti  2016 |
| --- | --- | --- | --- | --- | --- | --- | --- | --- | --- |
| (1) Define the source of information (survey, record review).(1) | yes | yes | yes | yes | yes | yes | yes | yes | yes |
| (2) List inclusion and exclusion criteria for exposed and unexposed subjects (cases and controls) or refer to previous publications.(1) | yes | yes | yes | yes | yes | yes | yes | yes | yes |
| (3) Indicate time period used for identifying patients.(1) | yes | no | yes | yes | no | yes | no | yes | yes |
| (4) Indicate whether or not subjects were consecutive if not population-based.(1) | yes | yes | yes | yes | yes | yes | yes | yes | yes |
| (5) Indicate if evaluators of subjective components of study were masked to other aspects of the status of the participants.(1) | unclear | unclear | unclear | unclear | unclear | unclear | unclear | unclear | unclear |
| (6) Describe any assessments undertaken for quality assurance purposes (e.g., test/retest of primary outcome measurements).(1) | no | yes | yes | yes | yes | yes | yes | yes | yes |
| (7) Explain any patient exclusions from analysis.(1) | no | no | yes | yes | yes | yes | yes | yes | yes |
| (8) Describe how confounding was assessed and/or controlled.(1) | yes | yes | yes | yes | yes | yes | yes | yes | yes |
| (9) If applicable, explain how missing data were handled in the analysis.(1) | no | no | no | no | no | no | no | no | no |
| (10) Summarize patient response rates and completeness of data collection.(1) | yes | no | no | no | no | yes | no | no | yes |
| (11) Clarify what follow-up, if any, was expected and the percentage of patients for which incomplete data or follow-up was obtained.(1) | Yes | yes | yes | yes | yes | yes | no | yes | yes |
| Total (11) | 7 | 6 | 8 | 8 | 7 | 9 | 6 | 8 | 9 |

Table S1 Quality Assessment for Cross-sectional Studies (continued)

| Study  Item | Kim  2017 | Zhang  2017 | Wang  2017 | Wu  2017 | Huang  2018 | Li  2019 | Goto  2019 | Zou  2019 | Wada  2019 | Zhao  2019 | Cho  2020 |
| --- | --- | --- | --- | --- | --- | --- | --- | --- | --- | --- | --- |
| (1) Define the source of information (survey, record review).(1) | yes | yes | yes | yes | yes | yes | yes | yes | yes | yes | yes |
| (2) List inclusion and exclusion criteria for exposed and unexposed subjects (cases and controls) or refer to previous publications.(1) | yes | yes | yes | yes | yes | yes | yes | yes | yes | yes | yes |
| (3) Indicate time period used for identifying patients.(1) | yes | yes | yes | yes | yes | yes | yes | yes | yes | yes | no |
| (4) Indicate whether or not subjects were consecutive if not population-based.(1) | yes | yes | yes | yes | yes | yes | yes | yes | yes | yes | yes |
| (5) Indicate if evaluators of subjective components of study were masked to other aspects of the status of the participants.(1) | unclear | unclear | unclear | unclear | unclear | unclear | unclear | unclear | unclear | unclear | unclear |
| (6) Describe any assessments undertaken for quality assurance purposes (e.g., test/retest of primary outcome measurements).(1) | yes | yes | yes | yes | no | yes | yes | yes | no | yes | yes |
| (7) Explain any patient exclusions from analysis.(1) | yes | yes | yes | yes | yes | no | yes | yes | yes | yes | yes |
| (8) Describe how confounding was assessed and/or controlled.(1) | yes | yes | yes | yes | yes | yes | yes | yes | yes | yes | yes |
| (9) If applicable, explain how missing data were handled in the analysis.(1) | no | no | no | no | no | yes | no | no | no | no | no |
| (10) Summarize patient response rates and completeness of data collection.(1) | no | yes | no | no | yes | yes | no | no | no | no | no |
| (11) Clarify what follow-up, if any, was expected and the percentage of patients for which incomplete data or follow-up was obtained.(1) | yes | yes | yes | yes | yes | yes | yes | yes | no | no | no |
| Total (11) | 8 | 9 | 8 | 8 | 8 | 9 | 8 | 8 | 6 | 7 | 6 |
