# Supplementary material for: Association Between Self-Reported Snoring and Metabolic Syndrome: A Systematic Review and Meta-Analysis
Source: Front Neurol. 2020 Oct 2;11:517120. doi: 10.3389/fneur.2020.517120 (PMC7566901; doi:10.3389/fneur.2020.517120)
Supplement: Supplementary file 6 [file Table_2.doc]

| Study  Item | | Marrone 1998 | Sun 2011 |
| --- | --- | --- | --- |
| Selection | (1) Is the case definition adequate?(1) | 0 | 1 |
|  | (2) Representativeness of the cases.(1) | 1 | 0 |
|  | (3) Selection of Controls.(1) | 1 | 1 |
|  | (4) Definition of Controls.(1) | 1 | 1 |
| Comparability | (1) Comparability of cases and controls on the basis of the design or analysis.(2) | 2 | 2 |
| Exposure | (1) Ascertainment of exposure.(1) | 0 | 0 |
|  | (2) Same method of ascertainment for cases and controls.(1) | 1 | 1 |
|  | (3) Non-Response rate.(1) | 1 | 0 |
| Total (9) | | 7 | 6 |

Table S2 Quality assessment for Case-control Studies
