# Supplementary material for: Association Between Self-Reported Snoring and Metabolic Syndrome: A Systematic Review and Meta-Analysis
Source: Front Neurol. 2020 Oct 2;11:517120. doi: 10.3389/fneur.2020.517120 (PMC7566901; doi:10.3389/fneur.2020.517120)
Supplement: Supplementary file 7 [file Table_3.doc]

| Study  Item | | Lindberg  1998 | Hu  1999 | Elmasry  2000 | Delaimy  2002 | Renko  2005 | Kim  2007 | Wendy  2010 | Shin  2014 | Lee  2018 | Wei  2020 |
| --- | --- | --- | --- | --- | --- | --- | --- | --- | --- | --- | --- |
| Selection | (1)Representativeness of the exposed cohort.(1) | 0 | 0 | 0 | 0 | 1 | 1 | 1 | 1 | 1 | 1 |
|  | (2) Selection of the non exposed cohort.(1) | 1 | 1 | 1 | 1 | 1 | 1 | 1 | 1 | 1 | 1 |
|  | (3) Ascertainment of exposure.(1) | 0 | 0 | 0 | 0 | 0 | 0 | 0 | 1 | 0 | 0 |
|  | (4) Demonstration that outcome of interest was not present at start of study.(1) | 0 | 1 | 0 | 1 | 1 | 1 | 1 | 1 | 1 | 1 |
| Comparability | (1) Comparability of cohorts on the basis of the design or analysis.(2) | 2 | 2 | 2 | 2 | 2 | 2 | 2 | 2 | 2 | 2 |
| Outcome | (1) Assessment of outcome .(1) | 0 | 0 | 0 | 0 | 1 | 1 | 1 | 1 | 1 | 1 |
|  | (2) Was follow-up long enough for outcomes to occur.(1) | 1 | 1 | 1 | 1 | 1 | 1 | 1 | 1 | 1 | 1 |
|  | (3) Adequacy of follow up of cohorts.(1) | 1 | 0 | 1 | 1 | 1 | 1 | 1 | 1 | 1 | 1 |
| Total (9) | | 5 | 5 | 5 | 6 | 8 | 8 | 8 | 8 | 8 | 8 |

Table S3 Quality Assessment for Cohort Studies
