# Supplementary material for: Association Between Self-Reported Snoring and Metabolic Syndrome: A Systematic Review and Meta-Analysis
Source: Front Neurol. 2020 Oct 2;11:517120. doi: 10.3389/fneur.2020.517120 (PMC7566901; doi:10.3389/fneur.2020.517120)
Supplement: Supplementary file 8 [file Table_4.docx]

Table S4 Characteristics of included studies in this meta-analysis

| **No** | **Year** | **Author** | **Region** | **Study** | **Sample size** | **Female**  **(%)** | **Age** | **OR and 95%CI** | | | |  |
| --- | --- | --- | --- | --- | --- | --- | --- | --- | --- | --- | --- | --- |
| 1 | 1987 | Gislanson | Sweden,  European | Cross-sectional study | 4064 | 0 | 30-69 | **Hypertension:**0.7926 (0.6239-1.0068) | | | |  |
| 2 | 1994 | Koskenvuo | Finnish  European | Cross-sectional study | 3750 | 0 | 40-59 | **Hypertension:**1.22 (0.99-1.51) | | | |  |
| 3 | 1996 | Enright | US,  North American | Cross-sectional study | 5201 | 57 | ≥65 | **Diabetes:**1.34 (1.10-1.65) | | | |  |
| 4 | 1998 | Lindberg | Sweden,  European | Prospective cohort study | 2668 | 0 | 45.1±11.1 | **Hypertension:**1.8 (1.1-3.0) | | | |  |
| 5 | 1998 | Marrone | Italian,  European | Case-control study | 90 | 50 | NA | **Hypertension:**  Men: 1.11 (0.44-2.77)  Women: 1.42 (0.61-3.26) | | | |  |
| 6 | 1999 | Hu | US,  North American | Cross-sectional and  prospective cohort study | 73231 | 100 | 40-65 | **Hypertension:**  Cross-sectional:  reference: never (3116/18928*)  Occasional (11993/47419): 1.22 (1.16-1.27)  Regular (2042/6884): 1.43 (1.33-1.53) | | | Prospective cohort:  reference: never (1595/105700)  Occasional (5147/225981): 1.29 (1.22-1.37)  Regular (880/27247): 1.55 (1.42-1.70) | |
| 7 | 2000 | Elmasry | Sweden,  European | Prospective cohort study | 2504 | 0 | 30-69 | **Diabetes:** 1.06 (0.36-3.10) | | | |  |
| 8 | 2002 | Delaimy | US,  North American | Prospective cohort study | 69852 | 100 | 40-65 | **Diabetes:** Reference: no snoring (2751/18340)  Occasional (7226/45161): 1.22 (1.00-1.48)  Habitual (1143/6351): 1.63 (1.29-2.07) | | | |  |
| 9 | 2003 | Leineweber | Sweden,  European | Cross-sectional study | 300 | 100 | 30-60 | **MetS**: 4.50 (1.71-11.86) | | | |  |
| 10 | 2004 | Marchesini | Italian,  European | Cross-sectional study | 1890 | 77.9 | 46 (20-65) | **Hypertension:** 1.39 (1.12-1.71)  **Diabetes:** 1.96 (1.47-2.63) | **Abdominal obesity**:** 1.16 (1.12-1.20) | | |  |
| 11 | 2005 | Renko | Finland,  European | Prospective cohort study | 593 | 58.7 | 61-63 | **Diabetes:** 1.93 (1.04-3.57) | | | |  |
| 12 | 2005 | Shin | Korean,  Asian | Cross-sectional study | 4762 | 0 | 40-69 | **Diabetes:** 1.00 (0.78-1.30) | | | |  |
| 13 | 2006 | Cho | Korean,  Asian | Cross-sectional study | 9547 | 52.80 | 40-69 | **Hypertension:** Men: 1.2 (1.0-1.4)  Women: 1.2 (1.0-1.5)  **Glyceridemia:** Men: 1.1(0.9-1.2)  Women**:**1.0(0.8-1.2) | | **HDL:** Men: 1.1 (0.9-1.3)  Women: 1.0 (0.8-1.2)  **Diabetes:** Men: 1.2 (1.0-1.5)  Women: 1.3 (1.0-1.6) | |  |
| 14 | 2007 | Lindberg | Sweden,  European | Cross-sectional study | 6779 | 100 | 44.7±17.2 | **Hypertension:** 1.12 (0.91-1.38)  **Diabetes:** 1.36 (0.87-2.13) | | | |  |
| 15 | 2007 | Kim | Korean,  Asian | Prospective cohort study | 5453 | 49.94 | 40-69 | **Hypertension:** Women: 1.56 (1.07-2.27)  Men: 1.49 (1.08-2.05) | | | |  |
| 16 | 2009 | Valham | Sweden,  European | Cross-sectional study | 7905 | 51.20 | 53±13 | **Diabetes:** Women: 1.58 (1.02-2.44)  Men: 0.92 (0.64-1.33) | | | |  |
| 17 | 2010 | Roopa | southern India,  Asian | Cross-sectional study | 358 | 53 | 20-76 | **MetS**: 2.252 (1.298-3.906) | | | |  |
| 18 | 2010 | Wendy | Pittsbugh, metropolitan area American | Prospective cohort study | 812 | 67 | 45-74 | **MetS**: 1.78 (1.02-3.12)  **Hypertension:** 0.64 (0.30-1.39)  **Triglyceride:** 2.11 (0.85-5.26) | **HDL:** 1.92 (1.06-3.48)  **Diabetes:** 2.15 (1.09-4.24)  **Abdominal obesity:** 0.80 (0.32-1.98) | | |  |
| 19 | 2011 | Sun | China,  Asian | Case-control study | 1003 | 61 | 35-54 | **MetS**: Total-Reference: never (108/396)  Occasional (163/367): 1.45 (0.96-2.19)  Regular (159/240): 1.95 (1.18-3.20) | | | |  |
| 20 | 2011 | Sabanayagam | US,  North American | Cross-sectional study | 6522 | 48.8 | 20-85 | **Diabetes:** Reference: never (298/3158)  Occasional (184/1256): 1.67 (1.23-2.27)  Frequent (355/2180): 1.44 (1.16-2.27) | | | |  |
| 21 | 2012 | Sabanayagam | US,  North American | Cross-sectional study | 6122 | 50.1 | 44.63±0.46 | **MetS**: Total-Reference: 0-2/week (803/2952)  3-4/week (466/1188): 1.69 (1.42-2.00)  ≥5/week (1025/1982): 2.77 (2.38-3.24) | | | |  |
| 22 | 2012 | Kazman | African Americans,  North American | Cross-sectional study | 248 | 63 | 44.6 ±11.5 | **MetS**: 2.57 (1.40-4.71) | | | |  |
| 23 | 2014 | Ikeda | Japan,  Asian | Cross-sectional study | 3936 | 40.4 | 56.5±16.3 | **MetS**:1.89 (1.56-2.30)  **Hypertension:** 1.11 (0.91-1.35)  **Dyslipidemia:** 1.50 (1.27-1.77) | **Diabetes:** 1.00 (0.84-1.20)  **Abdominal obesity:** 1.90 (1.60-2.26) | | |  |
| 24 | 2014 | Shin | Korean,  Asian | Prospective cohort study | 7038 | 40.55 | ≥40 | **MetS**:  Total-Reference: never (1283/3700)  Rare (431/1052): 1.16 (0.99-1.35)  Occasional (503/1066): 1.34(1.15-1.57)  Habitual (640/1230): 1.31 (1.13-1.52)  **Hypertension:**  Total-reference: never (698/3700)  Rare (217/1052): 1.08 (0.93-1.24)  Occasional (270/1066): 1.28 (1.11-1.48)  Habitual (334/1230): 1.34 (1.17-1.54) | | **Elevated FBG:**  Total-reference: never (259/3700)  Rare (75/1052): 1.04 (0.90-1.21)  Occasional (83/1066): 1.19 (1.03-1.38)  Habitual (109/1230): 1.28 (1.12-1.48) | |  |
| 25 | 2015 | Wang | China,  Asian | Cross-sectional study | 56032 | 61.32 | ≥40 | **Hypertension:**  Reference: never (7784/16675)  Occasional (9047/18848): 1.08(1.02-1.13) Usual (8843/16686): 1.30 (1.23-1.37) | | **HbA_1c_:**  Reference: never (6375/16675)  Occasional (7162/18848): 0.98 (0.92-1.03) Usual (6674/16686): 1.05 (1.00-1.11) | |  |
| 26 | 2016 | Brockmann | Chilean,  South American | Cross-sectional study | 2147 | 58 | 27.9 ±7.6 | **MetS**: 2.13 (1.52-2.99) | | | |  |
| 27 | 2016 | Modesti | Italian  European | Cross-sectional study | 1608 | 55.66 | 41.7±10.2 | **Hypertension:** 1.29 (1.14-1.45) | | | |  |
| 28 | 2017 | Kim | Korean  Asian | Cross-sectional study | 72885 | 65.90 | 40-69 | **MetS**：  Women-Reference: never (4874/25590)  1-3/week (2205/6736): 1.34 (1.25-1.44)  4-5/week (657/1779): 1.40 (1.25-1.57)  6+/week (1448/3504): 1.45 (1.33-1.58)  Men-Reference never (2216/9555)  1-3/week (1709/5190): 1.63 (1.51-1.75)  4-5/week (598/1634): 1.85 (1.65-2.07)  6+/week (1398/3620): 2.07 (1.91-2.25)  **Hypertension:**  Women-Reference: never (8249/25590)  1-3/week (3148/6736): 1.31 (1.24-1.39)  4-5/week (882/1779): 1.34 (1.20-1.48)  6+/week (1821/3504): 1.32 (1.22-1.43)  Men-Reference: never (4613/9555)  1-3/week (2982/5190): 1.44 (1.35-1.55)  4-5/week (990/1634): 1.57 (1.44-1.75)  6+/week (2156/3620): 1.57 (1.45-1.70)  **Triglyceride:**  Women-Reference: never (5288/25590)  1-3/week (2020/6736): 1.25 (1.17-1.33)  4-5/week (567/1779): 1.25 (1.12-1.39)  6+/week (1199/3504): 1.27 (1.17-1.38)  Men-Reference: never (3428/9555)  1-3/week (2299/5190): 1.39 (1.30-1.49)  4-5/week (729/1634): 1.44 (1.29-1.60)  6+/week (1698/3620): 1.56 (1.45-1.69) | | **HDL:**  Women-Reference: never (8206/25590)  1-3/week (26376736): 1.11 (1.00-1.23)  4-5/week (740/1779): 1.21 (1.12-1.30)  6+/week (1592/3504): 1.27 (1.17-1.38)  Men-Reference: never (2011/9555)  1-3/week (1207/5190): 1.04 (0.96-1.13)  4-5/week (429/1634): 1.12 (0.99-1.27)  6+/week (1045/3620): 1.22 (1.11-1.34)  **Diabetes:**  Women-Reference: never (412725590)  1-3/week (1602/6736): 1.23 (1.14-1.31)  4-5/week (474/1779): 1.32 (1.18-1.48)  6+/week (999/3504): 1.33 (1.22-1.45)  Men-Reference: never (2998/9555)  1-3/week (1879/5190): 1.23 (1.14-1.32)  4-5/week (637/1634): 1.32 (1.19-1.48)  6+/week (1355/3620): 1.29 (1.19-1.40)  **Abdominal obesity:**  Women-Reference: never (19121/25590)  1-3/week (3313/6736): 1.81 (1.71-1.92)  4-5/week (934/1779): 1.98 (1.79-2.19)  6+/week (2164/3504): 2.89 (2.66-3.09)  Men-Reference: never (1990/9555)  1-3/week (1603/5190): 1.69 (1.57-1.83)  4-5/week (546/1634): 1.86 (1.66-2.09)  6+/week (1467/3620): 2.57 (2.37-2.79) | |  |
| 29 | 2017 | Zhang | China  Asian | Cross-sectional study | 10139 | 53.73 | ＞35 | **Dyslipidemia:**  BMI＜25kg/m^2^: 1.119 (0.984-1.272)  BMI≥25kg/m^2^：1.207 (1.067-1.365)  **HDL:**  BMI＜25kg/m^2^: 1.130 (0.960-1.330)  BMI≥25kg/m^2^: 1.167 (1.001-1.361) | | **Triglyceride:**  BMI＜25kg/m^2^: 1.058 (0.874-1.281)  BMI≥25kg/m^2^: 1.110 (0.966-1.277) | |  |
| 30 | 2017 | Wang | China  Asian | Cross-sectional study | 13592 | 66.56 | 56.8±7.9 | **Diabetes:** Reference: never (4205/4521)  Occasional (1209/5617): 1.1(0.9-1.4)  Habitual (1703/3454): 0.9 (0.8-1.1) | | | |  |
| 31 | 2017 | Wu | China  Asian | Cross-sectional study | 57704 | 58.36 | Men:  53.15±10.20  Women:  51.72±9.69 | **Diabetes:**  Men: 1.02 (0.99-1.06)  Reference: never (350/9860)  Sometimes (262/6374): 1.09 (0.92-1.29)  Frequent (361/7791): 1.07 (0.91-1.25) | | Women: 1.03 (1.00-1.05)  reference: never (957/19780)  Sometimes (455/7446): 1.04 (0.92-1.17)  Frequent (529/6451): 1.15 (1.02-1.19) | |  |
| 32 | 2018 | Lee | Korean  Asian | Prospective cohort study | 4954 | 53 | 50.8±8.5 | **Hypertension**:  Total-Reference: never (995/2109)  Occasional (1109/2186): 1.01(0.93-1.10)  Habitual (374/659): 0.95 (0.84-1.07) | | Men-Reference: never (448/864)  Occasional (584/1089): 1.02 (0.90-1.16)  Habitual (218/385): 0.95 (0.81-1.12)  Women-Reference: never (547/1245)  Occasional (525/1097): 0.95 (0.84-1.07)  Habitual (156/274): 0.86 (0.71-1.04) | |  |
| 33 | 2018 | Huang | China  Asian | Cross-sectional study | 4286 | 50.58 | 45.45±16.09 | **Hypertension**:  Reference: never (111/2249)  Occasional (161/1431): 1.57 (1.19-2.07)  Habitual (179/606): 2.69 (2.08-3.49)  **Diabetes:**  Reference: never (91/2249)  Occasional (122/1431): 1.46 (1.09-1.97)  Habitual (125/606): 2.39 (1.71-3.36) | | **Dyslipidemia:**  Reference: never (52/2249)  Occasional (85/1431): 1.98 (1.37-2.87)  Habitual (112/606): 4.79 (3.21-7.16) | |  |
| 34 | 2019 | Li | China  Asian | Cross-sectional study | 4298 | 13.89 | None | **MetS**: 1.38 (1.17-1.62)  **MetS**:  Total-Reference: never (375/1335)  Occasional (556/1420): 1.31 (1.09-1.56)  Habitual (544/1039): 1.50 (1.24-1.82)  **Hypertension**:  Reference: never (579/1335)  Occasional (647/1420): 1.02 (0.87-1.19)  Habitual (633/1039): 1.30 (1.09-1.56)  **Hypertriglyceridemia:**  Reference: never (431/1335)  Occasional (566/1420): 1.07 (0.91-1.27)  Habitual (553/1039): 1.36 (1.13-1.64) | | **HDL:**  Reference: never (368/1335)  Occasional (431/1420): 1.08 (0.90-1.29)  Habitual (349/1039): 1.16 (0.95-1.42)  **Diabetes:**  Reference: never (159/1335)  Occasional (200/1420): 1.09(0.85-1.37)  Habitual (234/1039): 1.39 (1.09-1.79)  **Abdominal obesity:**  Reference: never (883/1335)  Occasional (1128/1420): 1.52 (1.25-1.83)  Habitual (927/1039): 1.97 (1.51-2.56) | |  |
| 35 | 2019 | Goto | Japan  Asian | Cross-sectional study | 2021 | 64 | Never snore:  58.2±13.6  ≤2/week:  57.7±11.9  ≥3/week:  57.0±11.9 | **Hypertension**:  Total-Reference: never (137/460)  ≤2/week (133/323): 1.74 (1.23-2.47)  ≥3/week (208/462): 1.79 (1.29-2.48) | | Men-Reference: never (43/116)  ≤2/ week (67/134): 2.02 (1.14-3.58)  ≥3/ week (133/256): 1.91 (1.14-3.21)  Women-Reference: never (94/344)  ≤2/ week (66/189): 1.57 (1.01-2.46)  ≥3/ week (75/206): 1.68 (1.09-2.59) | |  |
| 36 | 2019 | Zou | China  Asian | Cross-sectional study | 866 | 41.57 | ≥18 | **MetS**:  Total: 2.328 (1.340-4.045)  Women: 2.382 (1.136-4.994)  Men: 1.615 (0.931-4.936)  **Hypertension**:  Total: 1.730 (1.130-2.650)  Women: 0.899 (0.501-1.613)  Men: 3.493 (1.748-6.979)  **Triglyceride:**  Total: 1.814 (1.097-2.998)  Women: 2.803 (1.146-6.856)  Men: 1.443 (1.097-2.998) | | **HDL:**  Total: 0.876 (0.615-1.249)  Women: 0.897 (0.551-1.460)  Men: 0.808 (0.477-1.368)  **Hyperglycemia:**  Total: 1.217 (0.709-2.092)  Women: 0.899 (0.429-1.899)  Men: 1.217 (0.736-3.911)  **Abdominal obesity:**  Total: 1.810 (1.063-3.083)  Women: 2.306 (1.245-4.270)  Men: 0.943 (0.335-2.653) | |  |
| 37 | 2019 | Wada | Japan  Asian | Cross-sectional study | 24837 | 52.88 | ≥65 | **Hypertension:**  Men-Reference: never (840/2229)  Sometimes (2886/6604): 1.18(1.06,1.30)  Every day (1343/2869): 1.26 (1.12,1.41)  Women-Reference: never (1666/4175)  Sometimes (3244/7423): 1.14(1.12,1.15)  Every day (767/1537): 1.30 (1.10,1.53) | | **Diabetes:**  Men-Reference: never (339/2229)  Sometimes (991/6604): 0.99 (0.90,1.09)  Every day (516/2869): 1.17 (1.06,1.29)  Women-Reference: never (338/4175)  Sometimes (765/7423): 1.27 (1.22,1.32)  Every day (203/1537): 1.63(1.56,1.71) | |  |
| 38 | 2019 | Zhao | China  Asian | Cross-sectional study | 1518 | 43.10 | 53.5 ± 13.7 | **Hypertension:**  Reference: never (259/853)  ≤3/week(117/305): 1.05 (0.78-1.42)  ＞3/week(174/360): 1.10 (0.82,1.47) | |  | |  |
| 39 | 2020 | Cho | Korean  Asian | Cross-sectional study | 2724 | 64.54% | 30-64 | **Diabetes:**  Reference: never (90/1582)  1-2/week (78/762): 1.50 (1.05-2.13)  3-4/week (55/495): 1.60 (1.13,2.31)  ＞4/week (83/534): 2.05 (1.48-2.83) | |  | |  |
| 40 | 2020 | Wei | China  Asian | Cohort study | 482413 | 58.85% | 30-79 | **Diabetes:**  Men: 1.12 (1.06-1.18)  Women: 1.14 (1.09-1.19) | |  | |  |

Note: *means the number of cases/subjects in each level of snoring frequency. **means an effect value from abdominal obesity to snoring frequency. Abbreviation: BMI, body mass index; CHD, coronary heart disease; CRP, C-reactive protein; EDS, excessive daytime sleepiness; FBG, fasting blood glucose; FHD, family history of diabetes; GGT, gamma glutamyl transferase; HC, hip circumference; HDL, high density lipoprotein; MD, major depression; NHANES, the National Health and Nutrition Examination Survey; PBG, 2h postload blood glucose; SBP, systolic blood pressure; T2DM, type 2 diabetes mellitus; TIA, transient ischemic attacks; TG, triglyceride; TC, total cholesterol; WC, waist circumference; WBC, white blood cell; WHR, waist-to-hip ratio; NCEP ATPⅢ: the National Cholesterol Education Program Expert Panel on Detection, Evaluation and Treatment of High Blood Cholesterol in Adults; IDF: the International Diabetes Federation; NHLBI/AHA: the National Heart, Lung, and Blood Institute/American Heart Association; FOS: the Framingham Offspring Study; JSIM: the Japanese Society of Internal Medicine.

Table S4 Characteristics of included studies in this meta-analysis (continued)

| **No** | **Definition of snoring** | **Definition of end-points (MetS, blood pressure, triglyceride, HDL, glucose or obesity)** | **Confounding adjusted** |
| --- | --- | --- | --- |
| 1 | Questionnaire answer: those who reported “1 (never)” and “2 (seldom)” were as non-snorers; those who reported “3 (sometimes)” as occasional snorers; and those “4 (often)” and “5 (very often)” as habitual snorers. | **Hypertensive:**  The participants were classified hypertension if they were attending regular medical check-ups for it. | age, BMI. |
| 2 | Questionnaire answer: those who reported “sometimes” “often” “almost always” as habitual snorers; those who reported “frequent” as occasional snorers; those who reported “never” as non-snorers. | **Hypertension:**  Ask the participants whether hypertension had ever been diagnosed by a doctor. | age, obesity, heavy alcohol use, physical inactivity at leisure time, dyspnoea, hostility, morning tiredness. |
| 3 | Questionnaire answer: “no” “yes”, those who reported “yes” were as habitual snorers; else as non-habitual snorers. | **Diabetes:**  History of diabetes, current use of insulin or oral hypoglycemic medication, fasting glucose ≥140mg/dl，or 2-hour postload glucose ≥200mg/dl. | age, being married. |
| 4 | Questionnaire answer: those who reported “1 (never)” “2 (seldom)” “3 (sometimes)” were as non-habitual snorers; those who reported “4 (often)” “5 (very often)” as habitual snorers. | **Hypertension:**  The subjects were classified as hypertensive if they reported attending regular medical check-ups for hypertension and/or answer “yes” to the question “Do you have high blood pressure?” | △BMI, age, BMI, smoking,  physical inactivity, alcohol dependence. |
| 5 | Questionnaire answer: “no” “yes”, those who reported “no” were as non-snores; else as snores. | **Hypertension:**  Hypertension was classified according to the WHO-ISH criteria as a BP level higher than 140mmHg systolic or 90mmHg diastolic. | none |
| 6 | Questionnaire answer: Snoring and snoring variables were categorized into “never” “regularly” “occasionally”, those who reported “never” were as non-snores; else as snores. | **Hypertension:**  Incident cases of hypertension were identified by self-reports of physician-diagnosed hypertension. | cross-sectional: smoking, BMI, waist circumference.  prospective cohort: BMI, waist circumference, the 1976 BMI and weight change, marital status, living arrangements. |
| 7 | Questionnaire answer: those who reported “1(never)” “2(seldom)” “3(sometimes)” were as non-habitual snorers; those who reported “4(often)” “5(very often)” as habitual snorers. | **Diabetes:**  Diabetes were defined as those who answered “yes” to the question “Do you have diabetes?” | age, △BMI, snoring and obesity,  smoking alcohol dependence,  physical inactivity. |
| 8 | Questionnaire answer: “never” “regularly” “occasionally” to the question “Do you snore?”, those who reported “never” were as non-snores; else as snores. | **Diabetes:**  Classic symptoms associated with an elevated plasma glucose level or no symptoms, but at least two elevated plasma glucose values on different occasions or treatment with hyperglycemia medication. | age, history of high cholesterol, history of high blood pressure, time period, smoking, body mass index (eight categories), physical activity,  alcohol use, postmenopausal hormone use, family history of diabetes, usual sleeping position, number of hours of sleep per day, and years of shift-work, waist: hip ratio. |
| 9 | Questionnaire answer: those who reported “never” “rarely” “some of the time” were as non-snorers; those who reported “most of the time” as snorers. | **MetS** (FOS): the presence of the two or more of the following components:   1. Fasting serum glucose level ≥7.0mmol/L; 2. Arterial blood pressure ≥140/90 mmHg; 3. Fasting serum triglycerides ≥1.7mmol/L and/or high-density lipoprotein (HDL) <1.05mmol/L; 4. Obesity (waist-hip-ratio ＞0.85 and/or BMI ≥28kg/m^2^). | age. |
| 10 | Snoring was specifically investigated systematically asking patients about previous reports by relatives of their sleeping pattern, occasional or habitual snoring, and age of first report, using standardized questions. | None | age, sex, BMI. |
| 11 | Questionnaire answer: those who reported snoring every or almost every night were classified as habitual snorers. | **Diabetes:**  Previously diagnosed DM, OGTT according to WHO criteria in 1998. | age, weight gain, smoking, alcohol  dependence, physical inactivity. |
| 12 | Questionnaire answer: habitual snorers was defined as a snoring frequency 4 days or more per week, and if otherwise, non-habitual snorers was defined. | **Diabetes:**  A diagnosis of diabetes was made if a fasting glucose level was greater than or equal to 126mg/dl or when post challenge levels were greater than or equal to 200mg/dl or when participants were receiving oral hypoglycemic agents or insulin therapy. | age, current alcohol consumption, current smoking, BMI, systolic blood pressure, high-density lipoprotein cholesterol, family history of diabetes. |
| 13 | Questionnaire answer: those who reported “never” were as non-snorers; those who reported “1-3/week” as simple snorers; those who reported “4-5/week” “6-7/week” as habitual snorers. | **Hypertension:**  Systolic blood pressure was ≥130mmHg or a diastolic blood pressure was ≥85mmHg, or when a study participant was receiving anti-hypertensive medication. | age, abdominal obesity, HDL,  triglyceride, high fasting glucose. |
| 14 | Questionnaire answer: those who reported “1=never” were as non-snorers; those who reported “2=seldom” “3=sometimes” “4=often” “5=very often” as snorers. | **Hypertension:**  The subjects were classified as having hypertension if they reported attending regular medical examinations for hypertension and/or answered “yes” to the question “Do you have high blood pressure?”. | BMI, alcohol dependency, physical activity, smoking status, excessive daytime sleepiness. |
| 15 | Questionnaire answer: those who reported “never” “occasionally” “sometimes (1-3/week)” were as non-habitual snorers; those who reported “often (4-6/week)” “every night” as habitual snorers. | **Hypertension：**  Hypertension was defined on the basis of blood pressure≥140/90 mmHg or the use of anti-hypertensive medications. | age, BMI, alcohol consumption,  smoking, exercise. |
| 16 | Questionnaire answer: “no” “yes” for the question “Do you snore while asleep?”, those who reported “no” were as non-snores; else as snores. | **Diabetes:**  Diabetes mellitus was defined as a positive answer to the question “Do you suffer from diabetes mellitus” | age, smoking, BMI, waist circumference. |
| 17 | Questionnaire answer: “no” “yes”, those who reported “no” were as non-snorers; those who reported “yes” as snorers. | **MetS** (NCEP ATPⅢ 2001)**:** the presence of the three or more of the following components:   1. Fasting glucose ≥100mg/dl [5.6mmol/liter, slightly below cutoff for impaired fasting glucose (IFG)]; 2. Blood pressure ≥130/85mmHg; 3. Triglycerides ≥150mg/dl (1.7mmol/liter); 4. high-density lipoprotein(HDL) cholesterol ＜40mg/dl (1.0mmol/liter) in men or ＜50mg/dl(1.3mmol/liter) in women; 5. Waist circumference ≥90cm in men or ≥80cm in women (abdominal obesity). | age, sex, family history of diabetes, physical activity, smoking, alcohol. |
| 18 | Those individuals who endorsed the symptom ≥3 times per week were as snores and others coded as non-snores. | **MetS** (NCEP ATPⅢ 2001)**:** the presence of the three or more of the following components:   1. fasting glucose ≥110mg/dl; 2. Systolic blood pressure (SBP) ≥130 mmHg, or diastolic blood pressure ≥85mmHg; 3. Triglycerides ≥150mg/dl; 4. HDL-C ＜40mg/dl for males or ＜50mg/dl for females; 5. Waist circumference ＞102cm for males or 88cm for females. | age, sex, race, marital status, smoking status, alcohol consumption, sedentary lifestyle, presence of clinically significant depressive symptoms. |
| 19 | Questionnaire: Do you snore during the last month with 3 levels (regularly, occasionally or never), those who reported “never” were as non-snores; else as snores. | **MetS** (NCEP ATPⅢ 2005)**:** the presence of the three or more of the following components:   1. fasting plasma glucose ≥5.6mmol/L; 2. Blood pressure ≥130/85 mmHg，or current use of anti-hypertensive medications; 3. Triglycerides ≥1.7mmol/L; 4. HDL cholesterol ＜1.03 mmol/L in men or ＜1.30 mmol/L in women; 5. Waist circumferences ≥90cm in men or ≥80cm in women. | age, sex, smoking alcohol drinking, physical activity, education, family history of chronic diseases, marital status, annual income, self-related health status, depressive symptoms, sleep quality and duration, BMI, inflammatory markers, adipokines. |
| 20 | Questionnaire answer: snoring and snoring variable were categorized into never or rare, occasional (3-4/week), and frequent (5 or more/week). | **Diabetes:**  Diabetes defined as a serum glucose ≥126mg/dl after fasting for a minimum of 8 hours, a plasma glucose ≥200mg/dl for those who fasted ＜8 hours before their NHANES visit, or glycosylated hemoglobin≥6.5%，self-reported physician-diagnosed diabetes or current use of oral hypoglycemic medication or insulin. | age, sex, ethnicity, education, smoking, alcohol, physical activity, BMI, depression, SBP, CRP, TC. |
| 21 | Snoring and snoring variables were categorized into 0-2 nights/week, 3-4 nights/week, and 5 or more nights/week. | **MetS** (NCEP ATPⅢ 2005)**:** the presence of the three or more of the following components:   1. hyperglycemia defined as a fasting serum glucose ≥100mg/dl or on drug treatment for elevated glucose; 2. High blood pressure, ≥130/85mmHg or use of BP medications; 3. Elevated blood triglycerides, ≥150mg/dl; 4. Low high-density lipoprotein cholesterol,＜40mg/dl in men and ＜50mg/dl in women; 5. Abdominal obesity, waist circumference ＞102cm in men and 88cm in women. | age, gender, race, education, smoking, alcohol intake, moderate physical activity, depression. |
| 22 | Questionnaire answer: those who reported “not during the past month” “less than once a week” were as non-snorers; those who reported “once or twice a week” “three or more times a week” as snorers. | **MetS** (NHLBI/AHA)**:** the presence of the three or more of the following components:   1. fasting glucose≥6.1mmol/L; 2. Blood pressure≥130/85mmHg; 3. Triglycerides≥8.3mmol/L; 4. HDL≥2.2mmol/L for males, or 2.8mmol/L for females; 5. Waist size＞88.9cm for males, or 101.6cm for females. | age, sex. |
| 23 | Questionnaire answer: “no” “yes”, those who reported “no” were as non-snorers; those who reported “yes” as snores. | **MetS** (JSIM)**:** the presence of abdominal obesity in addition two or more of the following components:   1. hyperglycemic, fasting blood glucose level was 110mg/dl or higher or if they were taking insulin or other medications for reduction of blood glucose; 2. Hypertensive, (i) mean systolic blood pressure 130mmHg or higher; or (ii) mean diastolic blood pressure 85mmHg or higher, or (iii) receiving medications of reduction of blood pressure; 3. **Dyslipidemia**, (i) serum triglyceride level 150mg/dl or higher, or (ii) HDL cholesterol level less than 40mg/dl, or (iii) receiving medications for reduction of the serum triglyceride level; 4. Abdominal obesity, waist circumference measurement of 85cm or larger for men and 90cm or larger for women. | age class, sex, smoking, drinking, exercise, skipping meals, eating between meals, eating out definitely feeling, psychological stress, abdominal obesity (hypertension as end points only) |
| 24 | Questionnaire answer: those who reported “no” as non-snorers; those who reported “＜1 day per month” “1-3 days per month” were as rare snorers; those who reported “1-3days per week” as occasional snorers; those who reported “≥4 days per week” as habitual snorers. | **MetS** (NCEP ATPⅢ 2005)**:** the presence of three or more of the following components:   1. elevated FBG, ≥100mg/dl or current use of medication for diabetes; 2. High blood pressure, SBP ≥130mmHg and/or DBP ≥85 mmHg or current use of medication for hypertension; 3. Hypertriglyceridemia, ≥150mg/dl; 4. Low HDL cholesterol,＜40mg/dl for men and ＜50mg/dl for women; 5. Abdominal obesity, waist circumference ≥90cm for men and ≥85 for women. | age, sex, education, marital status, job, smoking, regular exercise, sleep duration, CRP, WBC, GGT, BMI. |
| 25 | Questionnaire answer: “never” “usually” “occasionally”, those who reported “never” were as non-snores; else as snores. | **Hypertension:**  Hypertension was defined ≥140/90 mmHg or taking anti-hypertensive medications.  **Glucose metabolism:**  Good glycaemic control was defined as HbA1c < 53 mmol/mol (7.0%), and poor glycaemic control was defined as HbA1c ≥ 53 mmol/mol (7.0%) | age, sex, BMI, waist circumference, self-evaluated sleep quality, duration of diabetes, anti-diabetic treatment, smoking status, alcohol drinking status, physical activity, depression status. |
| 26 | Questionnaire answer: “no” “yes”, those who reported “no” were as non-snorers; those who reported “yes” as snorers. | **MetS** (NCEP ATPⅢ 2001)**:** the presence of three or more of the following components:   1. fasting glucose ≥110mg/dl; 2. Blood pressure ≥140/90 mmHg; 3. Triglycerides ≥150mg/dl; 4. Low-density lipoprotein ＞130； 5. Waist circumference ＞102cm for men or ＞88cm for women. | age, sex, BMI, apnea, somnolence,  asthma. |
| 27 | Questionnaire answer: “no” “yes”, those who reported “no” were as non-snorers; those who reported “yes” as snorers. | **Hypertension:**  Hypertension was defined as systolic BP ≥140mmHg and/or diastolic BP≥90 mmHg, or self-reported anti-hypertensive treatment. | age, sex, education, parental history of hypertension, alcohol use, over weight or obesity, diabetes, high total cholesterol, high total cholesterol, high triglycerides, Italian speaking, health insurance, sleepless after awaking. |
| 28 | Snoring and snoring variables were categorized into “never (those who reported “no” “very rarely”)” “1-3/month” “1-3/week” “4-5/week” “6+/week”: those who reported “never” were as non-snores; else as snores. | **MetS** (NCEP ATPⅢ 2005)**:** the presence of three or more of the following components:   1. fasting glucose ≥100mg/dl or drug treatment for elevated fasting glucose; 2. **Hypertension:** Systolic blood pressure ≥130，diastolic BP ≥85mmHg or drug treatment for elevated BP; 3. Triglycerides ≥150mg/dl or drug treatment for elevated triglycerides; 4. High density lipoprotein cholesterol ≤40 and ≤50mg/dl in men and in women, respectively; 5. Waist circumference ≥90 and ≥80cm for men and women, respectively. | age, BMI, education, occupation,  marital status, smoking (men only),  alcohol drinking, regular exercise, menopausal status (women only), sleep duration. |
| 29 | Questionnaire answer: “no” “yes”, those who reported “no” were as non-snorers; those who reported “yes” as snores. | **Dyslipidemia:** the presence of one or more of the following components:   1. **high TC**: ≥6.21mmol/L(240mg/dl); 2. **Low HDL-C**:＜1.03mmol/L(40mg/dl); 3. High LDL-C: ≥4.16mmol/L(160mg/dl); 4. **High TG**:＞2.26mmol/L(200mg/dl); | none |
| 30 | Questionnaire answer: “never” “occasionally (1-2/week)” “habitually (≥3/week)”, those who reported “never” were as non-snores; else as snores. | **Diabetes:**  Self-reported diabetes or newly-diagnosed diabetes defined by FBG ≥7.0mmol/L or PBG ≥11.1mmol/L. | age, sex, BMI, education, WC, HC, WHR, smoke, alcohol drinker, tea drinker, total cholesterol, serum triglycerides, high density lipoprotein cholesterol, low density lipoprotein cholesterol, depression status, cardiovascular disease, hypertension, family history of diabetes, sleep quality, duration of night sleep. |
| 31 | Questionnaire answer: “never” “sometimes” “frequency”, those who reported “never” were as non-snores; else as snores. | **T2DM:**  T2DM was define as either having self-reported physician diagnosis of diabetes with an onset of age 30 or later or having a non-fasting blood glucose level ≥11.1mmol/L or fasting blood glucose ≥7.0mmol/L but no prior diagnosis of diabetes. | age, sleep duration, socioeconomic status, educational attainment, marital status, household income, occupation, smoking status, alcohol consumption, total physical activity, BMI, WC, percentage body fat, medical conditions (other than T2DM including stroke/TIA, CHD, hypertension, MD, FHD), medications for sleep. |
| 32 | Questionnaire answer: those who reported “never” were as non-snorers; those who reported “occasionally” “1-3/week” as occasional snorers; those who reported “4-6/week” “almost every night” as habitual snorers. | **Hypertension:**  Incident hypertension was defined as the first occurrences at any follow-up examination where the participants had BP≥140/90mmHg or were being treated with anti-hypertensive medication. | age, sex, BMI, drinking and smoking habits, regular exercise, family history of hypertension,  triglyceride, HDL cholesterol, the presence of diabetes and cardiovascular disease. |
| 33 | Questionnaire answer: those who reported “1” were as non-snorers; those who reported “2 (1-2/week)” as occasional snorers; those who reported “3(3-4/week; snoring did not affect the sleep of bed partner)” “4(3-4/week; snoring affected the sleep of bed partner, or had sleep apnea for years)” as habitual snorers. | **Hypertension:**  A systolic blood pressure ≥140mmHg or a diastolic blood pressure ≥90 mmHg, or when the subject was receiving anti-hypertensive medication.  **Hyperglycemia:**  A fasting serum glucose level ≥6.1mmol/L, or a 2-h postprandial serum glucose level ≥7.8mmol/L, or when the subject was receiving oral hypoglycemic agents or insulin therapy.  **Dyslipidemia:**  A fasting serum triglyceride level ≥7.1mmol/L, or a fasting serum high-density lipoprotein cholesterol level ＜0.9mmol/L in men and ＜1.0mmol/L in women. | age, sex, smoking status, drinking status, EDS, other components of metabolic syndrome. |
| 34 | Questionnaire answer: never,  occasionally, habitually. Occasionally refers to 1-2 days per week; habitually refers to ≥3 days per week, those who reported “never” were as non-snores; else as snores. | **MetS** (IDF): the presence of three or more of the following components:   1. **Triglyceride:** elevated TG: ≥1.7mmol/L; 2. **High blood pressure**: ≥130/85mmHg; 3. T2DM or fasting plasma glucose: ≥5.6mmol/L; 4. **HDL:** Reduced HDL-C:＜1.0mmol/L for men and ＜1.3mmol/L for women; 5. WC:＞85cm and 80cm in Chinese men and women , respectively. | age, sex, current smoking, alcohol consumption, marital status, physical activity level, monthly income, work type, BMI. |
| 35 | Snoring and snoring variables were categorized into “never (those who reported “1-2/month” “1-2/week”)” “≤2/week” “≥3/week”, those who reported “never” were as non-snores; else as snores. | **Hypertension:**  Hypertension was defined as any of or a combination of the following: systolic blood pressure ≥140mmHg, diastolic blood pressure ≥90mmHg，or current treatment with anti-hypertensive medication. | age, sex, current drinking and smoking, physical activity, BMI. |
| 36 | Snoring intensity was evaluated using a 10 cm visual analogue scale (VAS) from 0 to 10: those who reported “0” were as non-snores; else as snores. | **MetS** (NCEP ATPⅢ 2005): the presence of three or more of the following components:   1. **elevated TG**: ≥1.7mmol/L; 2. **High blood pressure**: ≥130/85mmHg; 3. Elevated fasting glucose: ≥5.6mmol/L or on drug treatment for elevated glucose; 4. **Reduced HDL-C**:＜1.03mmol/L for men and ＜1.3mmol/L for women; 5. **WC**:＞90cm and ＞80cm in Chinese men and women ,respectively. | age, sex, smoking status, alcohol consumption. |
| 37 | Snoring variable were categorized into “never” “sometimes(1-6/week)””every night”. | **Diabetes:**  A self-reported history of diabetes was defined as having undergone treatment or having unresolved sequela of those conditions at the time of survey completion.  **Hypertension:**  None | age, BMI, smoking index, alcohol, airway symptoms (wheezing, expectoration), systemic disorders (diabetes, heart diseases), income levels and education levels, and that after stratification with age categories, smoking status and BMI categories. |
| 38 | Snoring variables were categorized into “never” “≤3/week” and “＞3/week”. | **Hypertension:**  Hypertension was defined as blood pressure of at least 140/90 mmHg, or taking antihypertension treatment currently. | age, sex, and income plus smoking, alcohol intake, tea drinking, salt intake, physical activity, family history of hypertension, BMI, waist-to-hip ratio. |
| 39 | participants were inquired whether they  are a habitual snorer. If affirmative, they were subsequently asked to rate their snoring frequency: never or rare, seldom (1  to 2 time/month), sometimes (1 to 2 time/week), often (3 to 4  time/week), or frequent (almost every night). | **T2DM:**  Type 2 diabetes mellitus was defined as fasting glucose level ≥126 mg/dL, HbA1c ≥6.5%, a self-reported physician-diagnosis, or current use of oral glucose-lowering drugs or in­sulin injection. | age, sex, BMI, hypertension prevalence, education level, household income, longest held occupation, current smoking and drinking status, physical activity. |
| 40 | participants were asked whether they snored during sleep, and the options were:  (1) Yes, often; (2) Yes, sometimes; (3) Never/I do not know. In the present analysis, participants with the first option were categorized as habitual snorers while the rest of all were non-snorers. | **T2DM:**  A random glucose level ≥11.1 mmol/L or a fasting glucose level ≥7.0 mmol/L. | age, education, household income, marital status, smoking status, alcohol intake, physical activity, family history of diabetes, prevalent hypertension, prevalent stroke or transient ischemic attack, prevalent coronary heart disease, menopausal status (only for women), sleep duration, daytime napping, fresh vegetables, fresh fruit, red meat consumption, baseline BMI, baseline WC. |
