# Supplementary material for: Association Between Self-Reported Snoring and Metabolic Syndrome: A Systematic Review and Meta-Analysis
Source: Front Neurol. 2020 Oct 2;11:517120. doi: 10.3389/fneur.2020.517120 (PMC7566901; doi:10.3389/fneur.2020.517120)
Supplement: Supplementary file 9 [file Table_5.docx]

Table S5 Subgroup and meta-regression analysis for association between snoring and hypertension

| subgroups | | number | OR (95%CI) | model | *t* value | *p* value |
| --- | --- | --- | --- | --- | --- | --- |
| sex | men  women  men&women | 6  4  5 | **1.24 (1.10-1.39)**  **1.19 (1.03-1.36)**  **1.24 (1.13-1.35)** | fixed  fixed  random | 0.20 | 0.85 |
| study type | cross sectional  case control  cohort | 10  2  3 | **1.21 (1.13-1.29)**  **1.25 (0.37-2.12)**  **1.56 (1.21-1.91)** | fixed  fixed  fixed | 2.07 | 0.06 |
| region | Asian  others | 6  9 | **1.22 (1.10-1.34)**  **1.22 (1.13-1.34)** | fixed  fixed | -0.18 | 0.86 |
| quality | high  median or low | 8  7 | **1.24 (1.13-1.36)**  **1.22 (1.11-1.36)** | fixed  fixed | 0.63 | 0.54 |
| adjustment for confounders smoke | yes  no | 7  8 | **1.26 (1.16-1.35)**  **1.17 (1.04-1.31)** | fixed  fixed | -0.12 | 0.91 |
| adjustment for confounders alcohol | yes  no | 9  6 | **1.24 (1.10-1.38)**  **1.23 (1.13-1.32)** | fixed  fixed | 0.21 | 0.839 |
| adjustment for confounders BMI | yes  no | 9  6 | **1.18 (1.04-1.33)**  **1.25 (1.16-1.32)** | fixed  fixed | 0.51 | 0.62 |
| adjustment for confounders physical activity | yes  no | 7  8 | **1.27 (1.17-1.37)**  **1.17 (1.05-1.29)** | fixed  fixed | -0.73 | 0.47 |
| adjustment for confounders emotion | yes  no | 3  12 | **1.27 (1.18-1.36)**  1.11 (0.95-1.27) | fixed  fixed | -1.52 | 0.13 |
| adjustment for confounders sleep | yes  no | 3  12 | **1.23 (1.12-1.33)**  **1.24 (1.12-1.35)** | fixed  fixed | -0.30 | 0.77 |
