# Supplementary material for: Association Between Self-Reported Snoring and Metabolic Syndrome: A Systematic Review and Meta-Analysis
Source: Front Neurol. 2020 Oct 2;11:517120. doi: 10.3389/fneur.2020.517120 (PMC7566901; doi:10.3389/fneur.2020.517120)
Supplement: Supplementary file 10 [file Table_6.docx]

Table S6 Subgroup and meta-regression analysis for association between snoring and glycometabolism

| subgroups | | number | OR (95%CI) | model | *t* value | *p* value |
| --- | --- | --- | --- | --- | --- | --- |
| sex | men  women  men&women | 6  5  6 | **1.05 (1.02-1.08)**  **1.05 (1.03-1.07)**  **1.18 (1.04-1.32)** | random  random  random | 1.06 | 0.30 |
| study type | cross-sectional  cohort | 12  5 | **1.03 (1.01-1.05)**  **1.13 (1.10-1.71)** | random  fixed | 0.39 | 0.70 |
| region | Asian  others | 9  8 | **1.05 (1.03-1.07)**  **1.32 (1.14-1.50)** | random  fixed | -3.49 | **0.003*** |
| quality | high  median or low | 9  8 | **1.05 (1.03-1.07)**  **1.19 (1.05-1.32)** | random  fixed | -0.29 | 0.22 |
| adjustment for confounders smoke | yes  no | 12  5 | **1.05 (1.03-1.07)**  **1.32 (1.17-1.47)** | random  fixed | -2.73 | **0.02*** |
| adjustment for confounders alcohol | yes  no | 11  6 | **1.05 (1.03-1.07)**  **1.27 (1.13-1.40)** | random  random | -2.64 | **0.02*** |
| adjustment for confounders BMI | yes  no | 10  7 | **1.05 (1.03-1.07)**  **1.17 (1.05-1.29)** | random  fixed | -0.93 | 0.37 |
| adjustment for confounders physical activity | yes  no | 9  8 | **1.05 (1.03-1.05)**  **1.21 (1.10-1.33)** | random  fixed | -2.88 | **0.004*** |
| adjustment for confounders emotion | yes  no | 4  13 | **1.02 (0.84-1.19)**  **1.05 (1.04-1.07)** | random  random | -0.24 | 0.815 |
| adjustment for confounders sleep | yes  no | 5  12 | **1.05 (1.03-1.07)**  **1.15 (1.06-1.25)** | random  fixed | -1.65 | 0.11 |

*means the *p* value is statistically significant.
