# Supplementary material for: Association Between Self-Reported Snoring and Metabolic Syndrome: A Systematic Review and Meta-Analysis
Source: Front Neurol. 2020 Oct 2;11:517120. doi: 10.3389/fneur.2020.517120 (PMC7566901; doi:10.3389/fneur.2020.517120)
Supplement: Supplementary file 11 [file Table_7.docx]

Table S7 Subgroup and meta-regression analysis for association between snoring and dyslipidemia

| subgroups | | number | OR (95%CI) | model | *t* value | *p* value |
| --- | --- | --- | --- | --- | --- | --- |
| lipid parameter | HDL  triglyceride  dyslipidemia | 4  4  3 | **1.09 (1.00-1.18)**  **1.08 (1.00-1.17)**  **1.21 (1.12-1.31)** | fixed  random  random | 1.26 | 0.24 |
| study type | cross-sectional  cohort | 2  9 | **1.12 (1.06-1.19)**  1.96 (0.90-3.03) | random  fixed | 1.90 | 0.09 |
| region | Asian  others | 9  2 | **1.12 (1.06-1.19)**  1.96 (0.90-3.03) | random  fixed | -1.90 | 0.09 |
| quality | high  median or low | 7  4 | 1.06 (0.97-1.15)  **1.20 (1.10-1.29)** | fixed  random | 1.80 | 0.11 |
| adjustment for confounders smoke | yes  no | 5  6 | **1.31 (1.12-1.49)**  **1.10 (1.04-1.17)** | random  fixed | 2.59 | **0.03*** |
| adjustment for confounders alcohol | yes  no | 5  6 | **1.31 (1.12-1.49)**  **1.10 (1.04-1.17)** | random  fixed | 2.59 | **0.03*** |
| adjustment for confounders BMI | yes  no | 2  9 | **1.16 (1.06-1.26)**  **1.11 (1.02-1.19)** | fixed  random | -0.08 | 0.941 |
| adjustment for confounders physical activity | yes  no | 3  8 | **1.52 (1.28-1.77)**  **1.10 (1.03-1.16)** | fixed  fixed | 3.55 | **0.01*** |
| adjustment for confounders emotion | yes  no | 3  8 | **1.52 (1.28-1.77)**  **1.10 (1.03-1.16)** | fixed  fixed | 3.55 | **0.01*** |
